# SMARCAL1 is a targetable synthetic lethal therapeutic vulnerability in ATRX-deficient gliomas that use alternative lengthening of telomeres

**DOI:** 10.1093/neuonc/noaf300

**Published:** 2026-01-10

**Authors:** Alexandrea Brown, Laura M Strickland, Elise N Erman, Christopher J Pirozzi, Justin T Low, Bill H Diplas, Emiley Gibson, Mariah Shobande, Taher Khambati, Marharyta Krylova, Heng Liu, Roger E McLendon, Zachary J Reitman, Stephen T Keir, Lee Zou, David M Ashley, Matthew S Waitkus

**Affiliations:** The Preston Robert Tisch Brain Tumor Center, Duke University Medical Center, Durham; Department of Neurosurgery, Duke University Medical Center, Durham; The Preston Robert Tisch Brain Tumor Center, Duke University Medical Center, Durham; Department of Neurosurgery, Duke University Medical Center, Durham; The Preston Robert Tisch Brain Tumor Center, Duke University Medical Center, Durham; Department of Neurosurgery, Duke University Medical Center, Durham; The Preston Robert Tisch Brain Tumor Center, Duke University Medical Center, Durham; Department of Pathology, Duke University Medical Center, Durham; The Preston Robert Tisch Brain Tumor Center, Duke University Medical Center, Durham; Department of Neurosurgery, Duke University Medical Center, Durham; The Preston Robert Tisch Brain Tumor Center, Duke University Medical Center, Durham; Department of Neurosurgery, Duke University Medical Center, Durham; The Preston Robert Tisch Brain Tumor Center, Duke University Medical Center, Durham; Department of Neurosurgery, Duke University Medical Center, Durham; Department of Neurosurgery, Duke University Medical Center, Durham; The Preston Robert Tisch Brain Tumor Center, Duke University Medical Center, Durham; Department of Neurosurgery, Duke University Medical Center, Durham; The Preston Robert Tisch Brain Tumor Center, Duke University Medical Center, Durham; Department of Neurosurgery, Duke University Medical Center, Durham; Department of Pathology, Duke University Medical Center, Durham; The Preston Robert Tisch Brain Tumor Center, Duke University Medical Center, Durham; Department of Pathology, Duke University Medical Center, Durham; The Preston Robert Tisch Brain Tumor Center, Duke University Medical Center, Durham; The Preston Robert Tisch Brain Tumor Center, Duke University Medical Center, Durham; Department of Neurosurgery, Duke University Medical Center, Durham; Department of Pharmacology and Cancer Biology, Duke University Medical Center, Durham; The Preston Robert Tisch Brain Tumor Center, Duke University Medical Center, Durham; Department of Neurosurgery, Duke University Medical Center, Durham; The Preston Robert Tisch Brain Tumor Center, Duke University Medical Center, Durham; Department of Neurosurgery, Duke University Medical Center, Durham

**Keywords:** telomeres, synthetic lethality, ALT, replication stress, IDH-mutant astrocytoma

## Abstract

**Background:**

Approximately 10% of cancers achieve replicative immortality through a telomerase-independent mechanism of telomere maintenance, termed Alternative Lengthening of Telomeres (ALT). ALT is particularly prevalent in certain subtypes of malignant gliomas, such as *IDH*-mutant astrocytoma and pediatric glioblastoma, and frequently co-occurs with *ATRX* (ATRX chromatin remodeler) inactivating mutations. Although ALT is an adaptive mechanism through which cancer cells achieve proliferative immortality, the elevated levels of replication stress observed in ALT tumors constitute a potential therapeutic vulnerability.

**Methods:**

Leveraging CRISPR/Cas9 screening data from the Cancer Dependency Mapping Project, coupled with patient-derived cell lines and xenografts, we identified SMARCAL1 as a novel synthetic lethal vulnerability in ATRX-deficient glioma models that engage ALT. Using complementary molecular assays for DNA damage, telomere maintenance, and telomeric replication stress, we define the mechanisms underlying cytotoxicity induced by SMARCAL1 depletion in ALT-positive glioma cells.

**Results:**

Our data demonstrate the annealing helicase SMARCAL1 is a highly specific synthetical lethal vulnerability in cancers that use ALT. SMARCAL1 localizes to ALT-associated PML (Promyelocytic leukemia protein) bodies in ALT-positive glioma cell lines, including *IDH*-mutant astrocytomas. SMARCAL1 depletion, via doxycycline-induced RNAi, led to a hyperactivation of the ALT phenotype, high levels of DNA double-strand breaks in G2 phase, and cell death via mitotic catastrophe. In mice bearing intracranial xenografts derived from high-grade *IDH*-mutant astrocytoma, inducible SMARCAL1 depletion prolonged animal survival.

**Conclusions:**

Our findings demonstrate that the molecular processes orchestrating ALT-mediated telomere maintenance constitute a targetable synthetic lethal vulnerability that can be exploited by SMARCAL1 inhibition, thus supporting the future development of small molecule inhibitors of SMARCAL1 as anti-cancer therapeutics.

Key Points

- SMARCAL1 depletion causes ALT hyperactivation, DNA damage in G2 phase, and suppresses cell proliferation and xenograft growth.
- Future development of SMARCAL1 inhibitors may have significant potential for the treatment of ALT-positive gliomas.

Importance of the Study

Approximately 10% of all malignancies activate a cancer-specific mechanism of telomere maintenance termed Alternative Lengthening of Telomeres (ALT). Mechanistically, ALT involves the hijacking of DNA repair enzymes to use homologous recombination to extend telomeric DNA. Given its prevalence across various cancer types and the specificity of ALT as a process unique to cancer cells, the therapeutic targeting of ALT is a highly promising strategy for anti-cancer drug development. In this study, we identify the enzyme SMARCAL1 as a synthetic lethal therapeutic target in ATRX-deficient ALT-positive gliomas. We provide evidence that inhibition of SMARCAL1 activity, using genetic methods, induces high levels of DNA replication stress and DNA damage in glioma cells that use ALT. These studies identify SMARCAL1 as a promising drug target in ATRX-deficient ALT-positive cancers and support future development of small molecule inhibitors of SMARCAL1 for anti-cancer therapy.

Cancer cells acquire replicative immortality by activating telomere maintenance mechanisms, which prevent telomere shortening during successive rounds of cell division.^1^^,^^2^ While most cancers use the canonical enzyme telomerase to maintain telomere length, approximately 10% of all cancers use a telomerase-independent mechanism of telomere maintenance called Alternative Lengthening of Telomeres (ALT).^3^^,^^4^ ALT-positive tumors frequently harbor mutations in the *ATRX* (ATRX chromatin remodeler) gene, which encodes a histone H3.3 chaperone protein that catalyzes the deposition of H3.3 into telomeric chromatin.^5^^,^^6^ In the absence of ATRX, H3.3 deposition is compromised, which causes high levels of DNA replication stress at telomeres and leads to telomere extension via break-induced replication.^4^

While ALT is an adaptive mechanism by which telomerase-negative tumors enable replicative immortality, the molecular mechanisms and high levels of DNA replication stress associated with ALT constitute a vulnerability that can be targeted for anti-cancer therapy.^4^^,^^7–10^ Indeed, several published studies have shown that inhibiting enzymes involved in resolving DNA replication stress or catalyzing DNA repair can lead to excessive ALT activity, thus leading to intolerable levels of replication stress and genomic instability.^4^^,^^8^^,^^9^^,^^11^^,^^12^ In spite of this therapeutic potential, there are currently no approved anti-cancer therapies targeting ALT, nor has the ALT phenotype been successfully translated as a biomarker of response to clinically relevant anti-cancer agents.^2^

ALT is especially prevalent in tumors of mesenchymal origin, including several subtypes of adult and pediatric brain tumors.^13^ In malignant gliomas, telomere maintenance via ALT is strongly associated with *ATRX* loss-of-function mutations in *IDH*-mutant astrocytoma, pediatric supratentorial high-grade gliomas, 10% of diffuse midline gliomas with H3^K27M^ mutations, and a subset of adult *TERT* promoter wildtype glioblastoma (GBM).^6^^,^^13–15^ Although several studies have demonstrated that ATRX-deficient glioma cells are preferentially sensitive to distinct types of DNA damaging agents and DNA repair inhibitors,^7^^,^^10^^,^^16^^,^^17^ the strategy of stratifying patients by ALT status has not yet been translated into clinical development for glioma therapy. An improved understanding of ALT-associated therapeutic vulnerabilities in translational models is needed to identify novel therapeutic targets and inform the rational design of therapeutic strategies targeting ALT as an Achilles’ heel of ATRX-deficient gliomas.

Published studies from our laboratory and others have demonstrated that the annealing helicase SMARCAL1 resolves DNA replication stress in a variety of contexts, including replication fork stalling, ALT-associated telomere replication, and the removal of R-loops to prevent replication-transcription collisions.^12^^,^^14^^,^^18–21^ In this study, we provide evidence that SMARCAL1 is a highly specific synthetic lethal vulnerability in ATRX-deficient cancers that use ALT relative to non-ALT cells. In patient-derived glioma models, we found that ATRX-deficiency and the ALT phenotype correlated with significantly increased baseline levels of DNA replication stress and resulting DNA damage, including telomeric DNA double-strand breaks (DSBs). We provide evidence that inducible SMARCAL1 depletion exacerbates telomeric replication stress and increases the abundance of DNA DSBs in ALT-positive glioma cells, while SMARCAL1 depletion has little to no effect in telomerase-positive glioma cells. Mechanistically, depletion of SMARCAL1 in ALT-positive cells leads to hyperactivation of the ALT phenotype, excessive telomeric replication stress, DNA damage in G2 phase, and mitotic cell death. These studies identify SMARCAL1 as a promising drug target in ATRX-deficient ALT-positive cancers and support future development of targeted small molecule inhibitors of SMARCAL1 for anti-cancer therapy.

## Materials and Methods

### Cell Culture

D645MG, TM-31, D2363, NP5, LN229, KNS42, and LN18 cells were cultured in DMEM/F12 media supplemented with 10% FBS (Millipore Sigma, Cat# F2242-500ML, tetracycline free). TM-31 cells were shared by the Riken Bioresource Center under an MTA to Duke University. NP5 cells were received from the Japanese Collection of Research Bioresources (JCRB) cell biorepository under an MTA. D645MG and D2363MG cell lines were established from pleomorphic xanthoastrocytoma xenografts at Duke University.^22^ 08-0537, BT142, TS603, 12-0160, and 13-0302 were cultured as neurospheres in neural stem cell proliferation medium as previously described.^18^ Patient-derived neural stem cell cultures were passaged using the NeuroCult NS-A Proliferation Kit (Stemcell Technologies, Catalog # 05751) supplemented with 20 ng/ml human recombinant EGF (Stemcell Technologies, Catalog # 78006.2) and 10 ng/ml human recombinant bFGF (Stemcell Technologies, Catalog # 78134.1). Cells were routinely dissociated by mechanical trituration to create single cell suspensions and then passed through a 70-micron mesh filter and then seeded at 5 × 10^4^ viable cells per cm^2^. In some cases, Accutase digestion was used to fully dissociate neurospheres to achieve single-cell suspensions. To conduct adherent cell culture studies of neural stem-like glioma cells, cell culture plates and chamber slides were laminin-coated overnight in PBS containing Ca2+ and Mg2+ at a concentration of 1-2 ug/ml. For inducible knockdown of SMARCAL1 via RNAi, cells were transduced for 24 h with 5-10 MOI of lentiviral particles using the vector pLKO.1 Tet-on. After 24 h, media was changed and refreshed, and cells were expanded for at least 48 h prior to selection by puromycin. SMARCAL1-targeting and non-targeting sequences are listed in Supplemental Methods Table S1.

### Immunoblotting

Cells were lysed in RIPA buffer (Millipore Sigma, Catalog # R0278) with 1X protease inhibitor cocktail (Thermo Scientific, Catalog # 78430). Protein lysates containing 20 μg total protein were loaded and resolved using (4-12%) NuPAGE Bis-Tris gradient gel. Gels were soaked in protein transfer buffer (48 mM Tris, 39 mM glycine, 20% methanol, 0.0375% SDS) and transferred to a PVDF membrane using a BioRad Mini Tran-Blot transfer cell. After transfer to PVDF membranes, membranes were blocked with either Pierce TBST Protein-Free blocking buffer (Catalog # 37571) or 5% BSA in 1 × TBST and blotted with antibodies diluted in blocking buffer. Antibodies and blocking buffers used are detailed in Supplemental Methods Table S2.

### Immunofluorescence-Fluorescence in Situ Hybridization (IF-FISH) co-Staining

IF-FISH experiments were performed as described previously described.^18^ Cells were fixed for 10 min with 2% formaldehyde and then incubated with primary antibodies in blocking buffer (1 mg/ml BSA, 3% goat serum, 0.1% Triton X-100, 1 mM EDTA) overnight at 4 °C. Slides were incubated with secondary antibodies against rabbit or mouse IgG, conjugated with AlexaFluor 488 or 594 (ThermoFisher, 1:100). Slides were washed with PBS and fixed with 2% formaldehyde for 10 min at room temperature. Following a series of sequential dehydration steps (70%, 95%, and 100% ethanol), cells were incubated with PNA probes (each 1:2000) TelC-Cy3 (PNA Bio, Catalog # F1002) or TelC-AlexaFlour-647 (PNA Bio, Catalog # F1013) in hybridizing solution, denatured at 70 °C for 5 min on a ThermoBrite instrument, and then incubated at room temperature for 2 h or overnight at 4 °C in a humidity chamber. Slides were washed with 70% formamide, 10 mM Tris-HCl, PBS, stained with 4′,6-diamidino-2-phenylindole (DAPI), and then cover-slipped and sealed. Slides were imaged on a Zeiss 780 upright confocal microscope, and images were analyzed using Zen software. For quantitation of foci and co-localization of IF-stained foci with telomeres, the CellProfiler program was used. IF-FISH staining of FFPE tissues was performed using heat induced antigen retrieval in EDTA pH 8.0 buffer. Slides were incubated at 84 °C for 5 min and hybridized with a TelC-AlexaFlour-647 (PNA Bio, Catalog # F1013) in hybridizing solution for 2 h at room temperature, followed by 2 formamide-based washes (70% formamide in distilled water and 10 mM Tris-HCl) and PBST washes. For immunofluorescence co-labeling, slides were blocked (1 mg/ml BSA, 3% goat serum, 0.1% Triton X-100, 1 mM EDTA), incubated overnight at 4 °C with rabbit anti-PML (CST #69789S) antibody, washed with PBST, and then incubated with secondary antibodies (Invitrogen #A11008). Slides were stained with DAPI, treated with an autofluorescence-quenching reagent (Vector Laboratories VWR Cat# 103881-336), and mounted with VECTASHIELD Vibrance Antifade ounting Medium (Vector Laboratories VWR Cat# H-1700-10).

### ALT Telomere Synthesis Assay

Cells were grown on 3-well chamber slides (Ibidi) and synchronized to late G2 phase with 5 μM Ro-3306 CDK1i (Selleck Chem, Catalog # S7747) for 24 h. The cells were pulse labeled with 10 μM 5-ethynyl-2'deoxyuridine (EdU) in culture media for 2 h, fixed for 10 min with 4% formaldehyde, washed in PBS containing 3% BSA, and incubated with a Click-iT EdU Cell Proliferation Kit (ThermoFisher, Catalog # C10337) for 30 min. Slides were incubated with primary antibodies in blocking buffer (1 mg/ml BSA, 3% goat serum, 0.1% Triton X-100, 1 mM EDTA) overnight at 4 °C, after which they were washed and incubated with fluorophore-conjugated secondary antibodies (ThermoFisher, 1:100). Slides were rinsed with PBS and fixed with 2% formaldehyde for 10 min at room temperature and then processed for telomere FISH and imaging as described for IF-FISH experiments.

### C-circle Assay

The c-circle assay was performed as previously described.^14^ Briefly, total DNA was isolated from glioma cells using the Qiagen All-Prep kit (Catalog # 80204). 50 ng of total DNA was then amplified via rolling circle amplification in the presence or absence of φ29 polymerase for 8 h at 30 °C in buffer containing 4 mM dithiothreitol, 1 × φ29 buffer, 0.2 mg/ml bovine serum albumin (BSA), 0.1% Tween, and 25 mM of dATP, dGTP, dCTP, and dTTP. Amplified products were then blotted onto a Hybond-N+ (GE Amersham) nylon membrane using a BioDot (Bio-Rad) apparatus under vacuum filtration. Blotted DNA on membranes was ultraviolet crosslinked twice at 1200J. Prehybridization and hybridization were performed using the TeloTAGGG telomere length assay (Roche). Detection was performed using a DIG-labeled probe on an iBright imager (Invitrogen).

### IncuCyte Cell Proliferation Assay

Cells were seeded at 1 × 10^5^ cells per well in a 6-well dish and allowed to adhere overnight. The next day, media was replaced with fresh media and cells were allowed to proliferate in full growth media for 48 h. On day 3 after seeding, media was changed again to full growth media containing 1 μg/ml doxycycline, after which media was changed with fresh doxycycline-media every 48 h. Phase contrast images were acquired after doxycycline treatment using the IncuCyte live-cell imaging apparatus to determine cellular confluence between conditions.

### Cell Viability Assays

Cell viability assays were conducted using CellTiterGlo reagents according to the manufacturer’s instructions. Glioma cell lines were dissociated into single cell suspensions using Accutase or trypsin, filtered through a 70-micron filters, and plated into 96-well white-walled microplates (Corning, Catalog #3610) under adherent conditions. Cells were cultured in the presence or absence of doxycycline (1 μg/ml) for 6 or 10 days, and cell viability was measured using CellTiterGlo reagents. Data were acquired on a Tecan Infinite M200 Pro plate reader using a 1-s integration time, and luminescence values were normalized to the untreated control condition for each shRNA-expressing cell line.

### EdU-Pulse Labeling Immunofluorescence

Cells were grown on 3-well chamber slides (Ibidi) to sub-confluence and then pulse labeled with 10 μM 5-ethynyl-2'deoxyuridine (EdU) for 30 min. Cells were fixed for 10 min with 4% formaldehyde, washed in PBS containing 3% BSA, and incubated with a Click-iT EdU Cell Proliferation Kit (ThermoFisher, Catalog # C10337) for 30 min. Cells were then incubated with primary antibodies in blocking buffer (1 mg/ml BSA, 3% goat serum, 0.1% Triton X-100, 1 mM EDTA) overnight at 4 °C. After primary antibody incubation, the slides were incubated with goat secondary antibodies against rabbit or mouse IgG, conjugated with AlexaFluor 488 or 594 (ThermoFisher, 1:100). Slides were then washed PBS, stained with 4',6-diamidino-2-phenylindole (DAPI), and then cover-slipped and sealed. Slides were imaged on a Zeiss 780 upright confocal microscope, and images were analyzed using CellProfiler software. An anti-Cyclin A2 (Cell Signaling, Catalog # 67955) antibody is used as a marker for S-phase and G2 phase cells. Pan-nuclear EdU+ cells were considered S-phase cells, while the absence of pan-nuclear EdU staining and positive Cyclin A2 staining was used to denote G2 cells. All nuclei negative for both EdU and Cyclin A2 were considered in G1 phase.

### Flow Cytometry Analyses

For cell cycle analyses, cells were grown in 6-well tissue culture plate (Genesee) to sub-confluence. The cells were harvested using Accutase (Stemcell Tech, Catalog # 07922) for 5 min at 37 °C, washed with PBS, and fixed in ice-cold 70% ethanol for 1 h to overnight at 4 °C. Following fixation, cells were washed in PBS and incubated with FxCycle PI/RNase Staining Solution (ThermoFisher, Catalog # F10797) for 30 min at room temperature. Cells were sorted using a BD FACSCanto Flow Cytometry System and analyzed using FlowJo software. For chromatin-bound γH2AX flow cytometry, single-cell suspensions were treated with extraction buffer (0.2% Triton X-100) for 5 min on ice, followed by a wash with staining buffer (0.5% BSA in PBS), and then fixed and processed with the FoXP3 fixation kit (ThermoFisher Catalog# 00-5523-00). γH2AX primary antibody AlexaFluor 647 Conjugate (Cell Signaling #81330) was used at 1:250 dilution in staining buffer and data were acquired on a Cytek Aurora Spectral Flow Cytometer.

### Native Telomere FISH (ssTeloC) Assay

Cells were grown on 3-well chamber slides (Ibidi) to sub-confluence. The cells were fixed for 5 min with 2% formaldehyde diluted in PBS and were then incubated with RNase HII-A antibody (Santa Cruz, Catalog # SC-515475, 1:50) in blocking buffer (1 mg/ml BSA, 3% goat serum, 0.1% Triton X-100, 1 mM EDTA) for 1 h at 37 °C. After incubation, cells were rinsed with PBS for 5 min at room temperature. Following a series of sequential dehydration steps (70%, 95%, and 100% ethanol), cells were incubated with PNA probe (1:2000) TelG-AlexaFluor-488 (PNA Bio, Catalog # F1008) in hybridizing solution at room temperature for 2 h. Slides were then washed with 70% formamide, 10 mM Tris-HCl, PBS, stained with 4',6-diamidino-2-phenylindole (DAPI), and then cover-slipped and sealed. Slides were imaged on a Zeiss 780 upright confocal microscope, and images were analyzed using Zen software.

### Mouse Intracranial Injection

Glioma stem-like cells were transplanted into the right caudate nucleus of athymic nude mice (1 × 10^5^ cells per injection). Mice were placed in a Stoelting stereotactic injection device under isoflurane-induced anesthesia and given meloxicam (5 mg/kg) as an analgesic. An incision was made in the midline of the scalp over the position of the frontal cortex, and bregma was located visually. Cells were injected 2.5 mm right and 3.5 mm deep relative to Bregma using a 25-gauge needle, and the needle was raised ∼0.5 mm. After 1 min, the syringe was slowly removed to prevent efflux. The site of injection was then sealed with bone wax, bupivacaine was administered as a local anesthetic, and the incision was sealed with surgical glue. Animals were then monitored for the onset of neurological symptoms or a 20% loss of body weight and then euthanized using IACUC approved protocols.

### Subcutaneous Tumor Models

A sterile single cell suspension was prepared in a 1:1 solution of DPBS/Cells and Matrigel (Corning, Catalog # 356237). The cell suspension was loaded into a disposable 1 ml syringe with a 20-gauge needle. The tumor cell preparation was injected subcutaneously into the flank of mice at an inoculation volume of 100 μL with a cell inoculation of 1 × 10^6^ cells. Once a palpable tumor had formed, subcutaneous tumors were measured with hand-held calipers. Tumor volume was calculated according to the formula: [(width × length)^2^] × 0.5 = mm^3^ tumor volume. Mice were euthanized once tumors reached a volume of 2000 mm^3^ or by other humane endpoints.

### Statistical Analyses

For analyzing differences between doxycycline treated or untreated cells, an unpaired 2-tailed *t*-test was used. For assays with >2 groups, a one-way analysis of variance was used with correction for multiple comparisons by the Tukey method. Experiments in which the intensity of ALT biomarkers in the nucleus was positively skewed, a Kruskal-Wallis test was used along with Dunn’s method for adjusting for multiple comparisons. Errors bars of box and whiskers plots represent the 10-90^th^ percentiles of cell populations, while the upper and lower edges of boxes represent the 25^th^-75^th^ percentile. Horizontal line across the box represents the median. Survival analyses for in vivo animal studies were performed using the log-rank test. Longitudinal growth of xenografts was assessed by a mixed-effects model with Tukey post-hoc test for multiple comparisons. For figures and figure legends in the manuscript, the following ­annotation are used to denote varying thresholds of statistical significance: *P < 0.05. **P < 0.01. ****P < 0.0001. ns = non-significant.

## Results

### ALT-Positive Gliomas Exhibit Elevated Levels of Replication Stress and DNA DSBs

**Figure 1.**
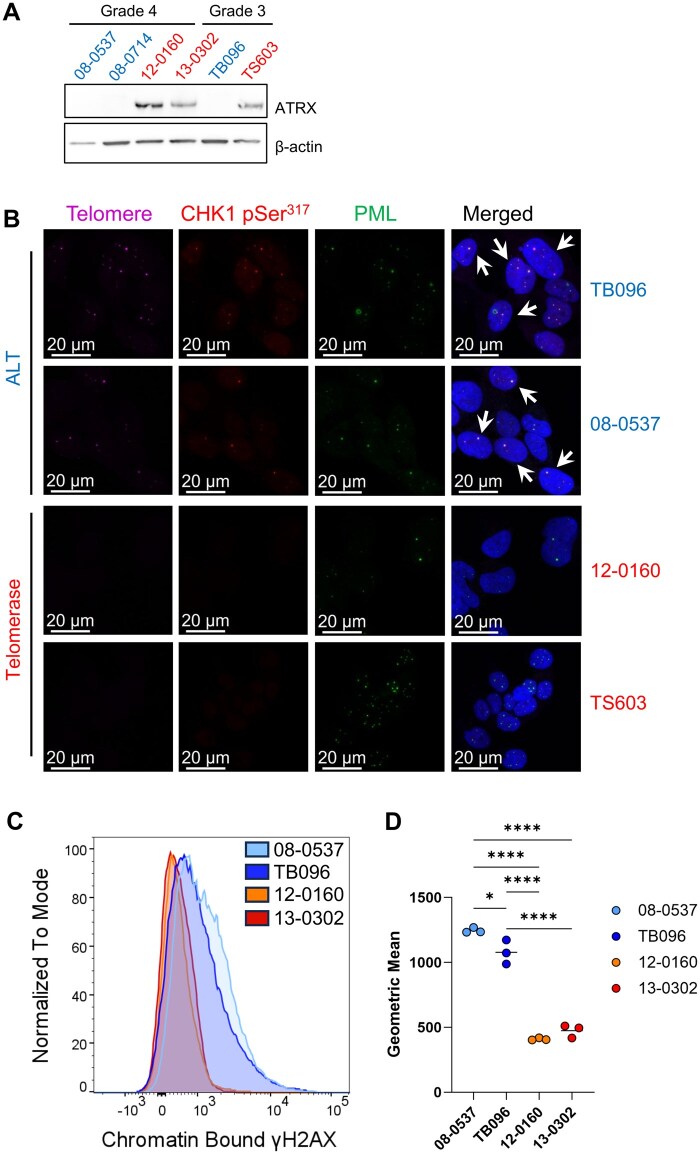
Alternative lengthening of telomeres-positive glioma cell lines exhibit high levels of DNA replication stress relative to telomerase+ glioma cell lines. (A) Western blot detection of ATRX protein expression in patient-derived glioma cell lines. Cell lines labeled in blue text represent ALT-positive cell lines, while red-labels indicate telomerase-positive cell lines. β-actin is used as a loading control for total protein. Data are representative of 3 independent experiments. (B) Confocal microscopy images of ALT-positive (TB096 and 08-0537) and telomerase-positive (12-0160 and TS603) cell lines stained with a TelC PNA probe, anti-PML antibody, and a CHK1 pSer^317^ antibody. (C) Flow cytometry data showing the extent of chromatin-bound γH2AX, a marker of DNA DSBs, between ALT-positive (TB096 and 08-0537) and telomerase-positive (12-0160 and 13-0302) cell lines. (D) Quantification of data shown in Figure 1C and analyzed using a one-way ANOVA and Tukey’s multiple comparisons test for differences between geometric means between conditions.

Alternative lengthening of telomeres-positive tumors are reported to exhibit higher levels of baseline DNA damage and replication stress relative to telomerase-positive tumors.^2^^,^^4^^,^^10^ We first examined the levels of specific markers of replication stress and resulting DNA double-strand breaks (DSBs) in a panel of ATRX-deficient ALT-positive patient-derived glioma cell lines (Figure 1A, Table S1). Alternative lengthening of telomeres-positive glioma cells exhibited characteristic ALT-associated PML bodies (APBs), indicated by the co-localization of telomeric foci within nuclear PML bodies (Figure 1B). Associated PML bodies in ALT-positive cells displayed activation of CHK1 signaling (Figure 1B), an indicator of the replication stress response initiated by TopBP1-ATR signaling axis.^23^^,^^24^ Phosphorylated CHK1 was absent in nuclear PML bodies within telomerase-positive cells (Figure 1B). Compared to telomerase-positive cells, ALT-positive cell lines displayed significantly higher levels of chromatin-bound γH2AX, indicating an elevated baseline level DNA DSBs (Figure 1C and D). Similarly, we observed γH2AX foci co-localized with APBs in TB096, BT142, and 08-0537 cells, indicating the presence telomeric DNA DSBs and ALT-mediated telomere maintenance (Figure S1A), consistent with ALT-positive cells undergoing break-induced replication of telomeres. The presence of nuclear single-stranded telomeric foci further indicated elevated telomeric replication stress and the presence of the ALT-phenotype in BT142 cells (Figure S1B), consistent with previous reports that BT142 is an ATRX-deficient ALT-positive *IDH*-mutant astrocytoma cell line.^25^^,^^26^

### Genetic Depletion of DNA Branchpoint Translocases Inhibits ALT-Positive Cancer Cell Growth

Having confirmed the presence of elevated baseline replication stress and the ALT phenotype in a panel of patient-derived ATRX-deficient glioma cell lines, we reasoned that the mechanism of break-induced telomere replication and high levels of ALT-associated DNA replication stress may render ATRX-deficient glioma cells vulnerable to the depletion of specific enzymes involved in DNA repair and the resolution of replication stress. To identify novel dependencies in ALT-positive cancer cells, we leveraged publicly available data from DepMap and identified a subset of ALT-positive cancer cell lines with established alterations in *ATRX* or *DAXX* from published literature.^27–33^ The G292 Clone A141B1 cell line was included, based on its established ALT status and presence of a *DAXX* fusion.^27^^,^^34^ Using DepMap genetic datasets, we identified 2 high-grade glioma cell lines, TM-31 and NP5, as candidate ALT-positive cell lines based on the presence of *ATRX* inactivating mutations (Table S2). We empirically validated TM-31 and NP5 as ALT-positive based on the absence of ATRX protein expression and the presence of APBs and single-stranded C-rich telomeric DNA (Figure S2A-C). Further, TM-31 cells exhibited *de novo* telomere synthesis at APBs (Figure S2D), as indicated by EdU staining co-localized with APBs using the ALT telomere synthesis assay (ATSA).^35^ In addition, we included the *IDH*-mutant astrocytoma TB096 cell line, established in previous studies,^36^ which has been profiled in the DepMap CRISPR dataset and is ATRX-deficient and ALT-positive ­(Figures 1A and B, S1A).

**Figure 2.**
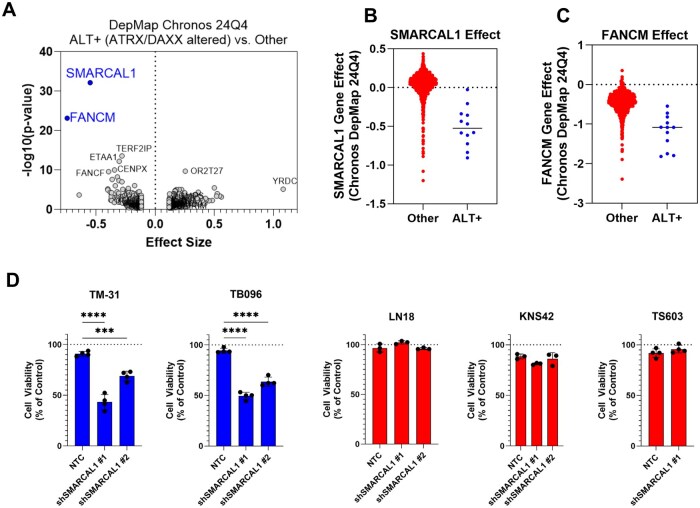
SMARCAL1 is an ALT-specific gene dependency in the Cancer Dependency Map dataset. (A) Volcano plot showing the differential gene dependency effects of ALT-positive cancer cell lines (*n* = 12) in the Cancer Dependency Map dataset versus all other cell lines in CRISPR/Cas9 screening dataset. Data are from the DepMap24Q4 release. (B) Cell line specific effects of SMARCAL1-targeting sgRNAs on cell lines within the DepMap dataset. (C) Cell line specific effects of FANCM-targeting sgRNAs on cell lines within the DepMap dataset. (D) ALT+ (blue) or telomerase+ (red) cell lines expressing non-targeting (NTC) or SMARCAL1-targeting shRNAs were seeded in 96-well plates and allowed to proliferate in the absence or presence of doxycycline for 6-days (TM-31) or 10-days (TB096, LN-18, KNS42, TS603). Cell viability was measured using CellTiterGlo and values compared relative to the minus-doxycycline controls (represented by dashed line). Differences between treatment conditions were assessed via one-way ANOVA with Dunnett’s test for multiple comparisons to control. TS603 data were analyzed with an unpaired 2-tailed *t*-test.

Using the DepMap CRISPR/Cas9 screening dataset, we compared gene dependencies in our empirically validated ALT-positive cell lines (*n* = 12) versus all other cell lines (*n* = 1166). Two genes, *SMARCAL1* and *FANCM*, exhibited the largest ALT-specific gene dependency effect (Dataset S1, Figure 2A). SMARCAL1 and FANCM are DNA branchpoint translocase enzymes that catalyze the reversal of stalled DNA replication forks, the removal of R-loops, and the resolution of telomeric replication stress.^20^^,^^37^^,^^38^ Importantly, FANCM inhibition has previously been shown to induce synthetic lethal effects in ALT-positive cancer cell lines.^8^^,^^9^ While *FANCM* deletion showed a larger detrimental effect in ALT-positive cells, *SMARCAL1* deletion suppressed cell proliferation in a highly ALT-specific manner (Figures 2B and C), exhibiting essentially no detrimental effects to non-ALT cells (Figure 2B, Dataset S2, S3). To validate these results from the DepMap dataset, we used doxycycline-inducible non-targeting shRNA or SMARCAL1-targeting shRNA in ALT-positive (TM-31 and TB096) and telomerase-positive (LN-18,^39^ KNS42,^40^ TS603^41^^,^^42^) glioma cell lines and measured the effect of inducible SMARCAL1 depletion on cell viability. SMARCAL1 depletion caused a loss of cell fitness in ALT-positive cell lines but did not significantly reduce fitness of telomerase-positive lines (Figure 2D), consistent with DepMap CRISPR/Cas9 screening data (Figure 2A and B). Notably, for both *SMARCAL1* and *FANCM*-dependent effects in the DepMap dataset, there was a negatively-skewed tail in the non-ALT cell line groups, suggesting that there are uncharacterized ALT-positive cell lines that are sensitive to SMARCAL1 depletion or that uncharacterized ALT-independent sources of DNA replication stress may sensitize cells to SMARCAL1 and FANCM depletion.

Other genes that trended toward ALT-specific gene dependency were TERF2IP (RAP1), a component of the telomeric shelterin complex^1^ (Figure S3A, Dataset S4), and CENPX, a FANCM-binding protein that promotes FANCM DNA-binding and fork reversal activity^43^ (Figure S3B, Dataset S5). In addition, several other components of the Fanconi Anemia core complex were identified as ALT-specific dependencies ­(Dataset S1 and S6), albeit with smaller effect sizes than FANCM, suggesting that loss of FANCM activity is more specifically detrimental to ALT-positive cells (Figures 2A, S3C). ATR and ETAA1, an ATR-activating protein, also demonstrated ALT-specific gene dependency effects (Figure S3D,E, Dataset S7, S8), consistent with published studies demonstrating that ALT-positive cells are highly vulnerable to ATR inhibition.^7^

### SMARCAL1 Localizes to APBs and Suppresses ALT Activity in ATRX-Deficient Glioma Cells

**Figure 3.**
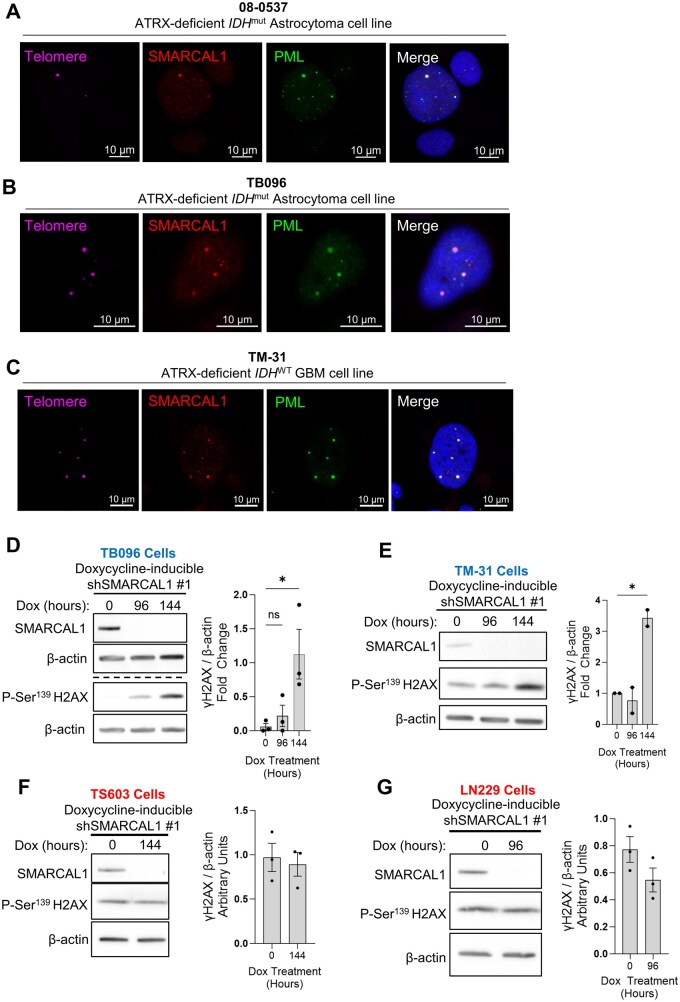
SMARCAL1 depletion causes increased DNA DSBs specifically in ALT-positive glioma (A) Confocal microscopy images of 08-0537 glioma cells stained by immunofluorescence-FISH using a TelC PNA probe and SMARCAL1 and PML antibodies. SMARCAL1 foci localize to APBs in APB+ nuclei. Images were acquired on a Zeiss 780 upright confocal microscope under 63× oil immersion. (B) TB096 cells stained and imaged as in (A). (C) TM-31 cells stained and as in (A). (D) TB096 transduced with doxycycline-inducible SMARCAL1-targeting shRNA were treated with doxycycline (1μg/ml) for 96 or 144 h, after which cells were lysed and processed for western blotting analysis of γH2AX, a marker of DNA DSBs. Densitometry measurements of western blot images were performed in ImageJ and analyzed via a One-Way ANOVA and Dunnett’s multiple comparisons test to assess differences from control. (E) Experiment performed as in (D) with TM-31 cells stably transduced with an inducible SMARCAL1-targeting shRNA. (E) TS603 and (F) LN229 cells, respectively, were stably transduced with an inducible SMARCAL1-targeing shRNA for 144 or 96 h. After doxycycline treatment, cells were lysed and γH2AX was analyzed via western blot. Densitometry was quantitated in ImageJ.

SMARCAL1 is reported to resolve replication stress at telomeres in both ALT-positive and telomerase-positive cells, but the therapeutic potential of SMARCAL1 inhibition has not been investigated.^12^^,^^19^ SMARCAL1 protein was expressed in both ATRX-deficient ALT-positive cell lines as well as telomerase-positive cell lines (Figure S4A). In a panel of 6 ALT-positive glioma cell lines, SMARCAL1 localized to APBs, suggesting that SMARCAL1 activity is involved in regulating ALT-associated replication stress (Figures 3A-C, S4B-D).

To define the potential of inhibiting SMARCAL1 for therapeutic purposes in ALT-positive gliomas, we established a panel of patient-derived glioma cell lines with stable expression of doxycycline-inducible shRNA targeting SMARCAL1. Inducible depletion of SMARCAL1 via RNAi led to increased DNA DSBs in ALT-positive lines after a 6-day treatment period with doxycycline (Figures 3D and E, S5A-C), which corresponds to an approximately 4-day depletion of SMARCAL1 protein (Figure S5C). Inducible SMARCAL1 depletion via RNAi did not cause detectable increases in DNA DSBs in telomerase-positive cells (Figures 3F and G, S5D), suggesting that the DNA-damaging effects of SMARCAL1 depletion are ALT-specific. Doxycycline treatment suppressed cellular proliferation in ALT-positive TB096 cells expressing SMARCAL1-targeting shRNA relative to cells expressing non-targeting shRNA (Figure S5E). Furthermore, doxycycline treatment led to an increase in nuclear γH2AX staining in TB096 cells expressing inducible SMARCAL1-targeting shRNA, but not in cells expressing non-targeting shRNA (Figure S5F). These results demonstrate that doxycycline was not a significant contributor to DNA DSBs in our inducible RNAi models and that loss of cellular fitness in ALT-positive cell lines is due to SMARCAL1 depletion.

### SMARCAL1 Activity Limits Replication Stress and DNA Damage in ATRX-Deficient ALT-Positive Glioma Cells

**Figure 4.**
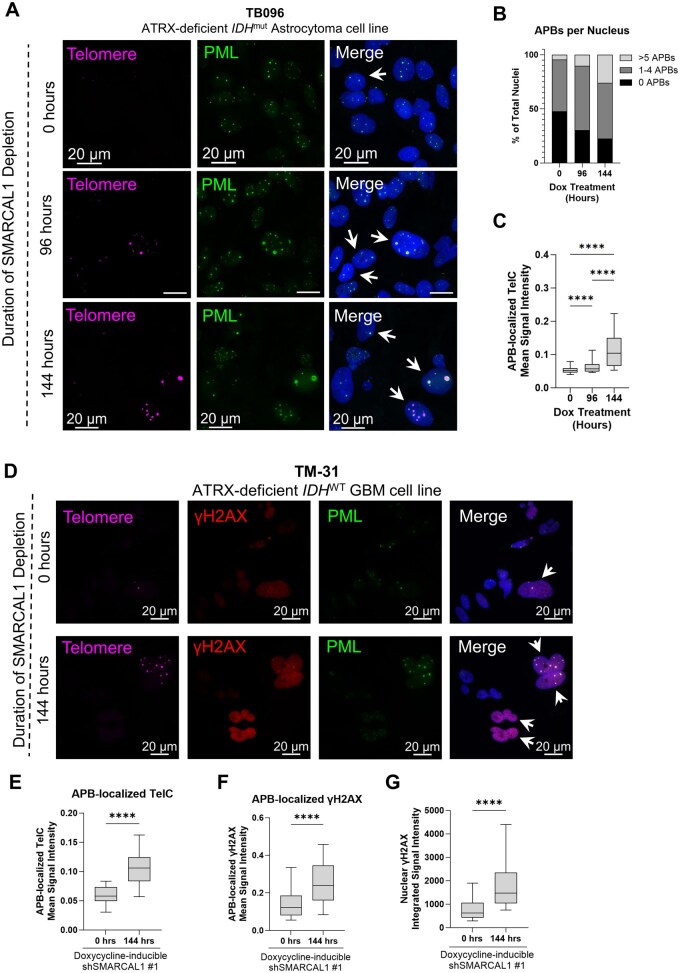
Depletion of SMARCAL1 leads to a hyper-ALT phenotype and DNA damage in ALT-positive cells. (A) ALT-positive TB096 cells (*IDH*-mutant astrocytoma) stably transduced with a doxycycline-inducible shRNA vector targeting SMARCAL1 were treated with doxycycline for 96 or 144 h. Following doxycycline treatment, cells were fixed and processed for IF-FISH to assess the abundance and intensity of APBs using a TelC PNA probe conjugated to Alexa-Fluor-647 and an anti-PML primary antibody detected using an anti-rabbit IgG Alexa-Fluor-488 secondary antibody. Images were acquired on a Zeiss 780 upright confocal microscope under 63× oil immersion. (B) Images in (A) were analyzed using Cell Profiler software to quantitate the abundance of APBs per nucleus. Differences in proportions across groups were analyzed using a chi-square test (*P* < 0.001). (C) Mean intensity of TelC signal in APBs in TB096 cells following inducible SMARCAL1 depletion was quantitated using Cell Profiler software. Data represent the quantitation of 2 independent experiments. Data were analyzed using the Kruskal-Wallis test with Dunn’s test for pair-wise comparisons. (D) ALT-positive TM-31 cells (*IDH*-wildtype GBM) stably transduced with a doxycycline-inducible shRNA vector targeting SMARCAL1 were treated with doxycycline (1μg/ml) for 144 h. After doxycycline treatment, cells were fixed for IF-FISH to assess the abundance and intensity of APBs using a TelC PNA probe conjugated to Alexa-Fluor-647, an anti-PML rabbit antibody, and an anti-γH2AX mouse antibody. Primary antibodies were detected using an anti-rabbit IgG Alexa-Fluor-488 and anti-mouse IgG Alexa-Fluor-594 secondary antibodies. Images were acquired on a Zeiss 780 upright confocal microscope under 63× oil immersion. (E) Confocal microscopy images were analyzed in Cell Profiler software to quantitate the TelC signal intensity APBs in APB+ nuclei. (F) γH2AX signal intensity at APBs in APB+ cells. (G) Total nuclear γH2AX signal intensity from cells treated as described in (A) above. For panels E-G, data are analyzed using an unpaired 2-tailed Mann-Whitney *U* test to assess differences between groups.

Given the ALT-specific gene dependency of SMARCAL1 ­(Figure 2A, B, and D), as well as the established role of SMARCAL1 and FANCM in replication fork reversal,^37^^,^^38^^,^^44^ we sought to understand the mechanism by which depletion of SMARCAL1 leads to DNA DSBs and the suppression of ALT-positive cell proliferation. We hypothesized that in the context of ATRX-deficiency, the reversal and stabilization of stalled replication forks effectively competes with break-induced replication of telomeres and SMARCAL1 activity is necessary to restrain excessive ALT activity to prevent intolerable levels of DNA damage and genomic instability. In ALT-positive glioma cells, inducible SMARCAL1 depletion led to an increase in the intensity and abundance of APBs in TB096 *IDH*-mutant astrocytoma cells and TM-31 GBM cells (Figure 4A-E). The increased intensity of APBs coincided with increased γH2AX staining at APBs and total nuclear γH2AX, suggesting that excessive replication stress was leading to DNA DSBs (Figure 4F-G). A characteristic phenotype of ALT-positive cells is the presence of telomeric c-circle DNA, which is generated during break-induced telomere replication in a BLM-dependent manner.^35^ Consistent with a role in suppressing ALT activity, inducible SMARCAL1 depletion caused an increase in telomeric c-circle DNA in D645MG and TB096 cells (Figure S6A,B) and increased the intensity of ssTeloC nuclear foci in TM-31 cells (Figure S6C,D).

### SMARCAL1 Depletion Leads to a hyper-ALT State and DNA Damage in G2 Phase

**Figure 5.**
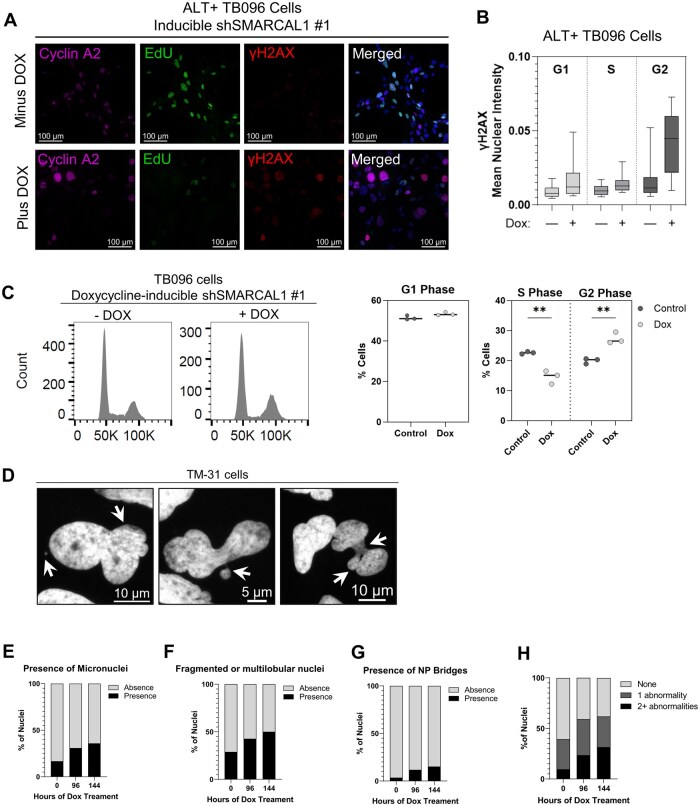
Inducible depletion of SMARCAL1 leads to extensive DNA damage in G2 phase and mitotic cell death. (A) TB096 cells stably expressing an inducible SMARCAL1-targeting shRNA vector were treated with doxycycline (1μg/ml) for 6 days, pulse labeled with EdU (10 μM) for 30 min, and then processed for immunofluorescence. (B) γH2AX staining intensity was quantitated on a nucleus-by-nucleus basis in G1 (EdU^-^/Cyclin A2^-^), S-phase (Edu^+^), and G2 (Edu^-^/Cyclin A2^+^) using Cell Profiler software. (C) Cell cycle analysis of TB096 cells treated with doxycycline for 6 days to inducibly deplete SMARCAL1. Differences between groups were assessed by an unpaired 2-tailed *t*-test and correction for multiple comparisons using the Holm-Šídák method. (D) Representative DAPI-stained nuclear morphologies observed in TM-31 cells after 6-days depletion of SMARCAL1 via doxycycline-inducible shRNA. Arrows denote micronuclei, multi-lobular nuclei, and chromatin bridges. (E-G) Quantitation of nuclear morphologies from DAPI-stained nuclei imaged under 63× oil immersion. (H) Quantitation of the percentage of nuclei exhibiting 2 or more nuclear abnormalities quantitated as in E-G. A chi-square test was used to assess differences in proportions across groups in E (*P* = .005), F (*P* = .007), G (*P* = .01), and H (*P* = .0006).

The above results suggested that SMARCAL1 activity is critical for limiting ALT-associated replication stress to prevent excessive DNA damage in ATRX-deficient ALT-positive glioma cells. Because ALT telomere synthesis occurs primarily in G2 phase of the cell cycle,^11^^,^^45^ we investigated the impact of SMARCAL1 depletion on DNA damage in asynchronous ALT-positive glioma cell lines across distinct phases of the cell cycle. To this end, we used an EdU-pulse labeling approach followed by immunofluorescence to detect G1 cells (EdU^-^/Cyclin A2^-^), S-phase cells (pan-nuclear EdU^+^), and G2 cells (pan-nuclear EdU^-^/Cyclin A2^+^) (Figure 5A). Using this assay, we found that SMARCAL1 depletion in TB096 and TM-31 cells led to mild increases in nuclear γH2AX staining in G1 and S-phase cells, whereas γH2AX was drastically increased in G2 cells following SMARCAL1 depletion (Figures 5B, S7A,B). Cell cycle analyses via flow cytometry showed that inducible SMARCAL1 depletion caused an enrichment of cells in the G2 phase of the cell cycle in TB096 *IDH*-mutant astrocytoma cell and TM-31 GBM cells, with a concomitant decrease in S-phase cells in both cell lines ­(Figures 5C, S7C,D).

We hypothesized that excessive replication stress and DNA damage in G2 phase would increase the frequency of aberrant mitotic progression in SMARCAL1 depleted cells, thus leading to mitotic cell death. To address this question, we quantified the abundance of nuclear morphological abnormalities indicative of mitotic catastrophe in TM-31 and TB096 cells that had been inducibly depleted of SMARCAL1 using RNAi for 6 days. We observed an increase in cells with micronuclei, nucleoplasmic bridges, and cells with fragmented or multi-lobular nuclei after SMARCAL1 depletion via RNAi (Figures 5D-H, S8A-E), suggesting that failure to resolve replication stress in G2/M phase was leading to mitotic catastrophe in these cells.

### SMARCAL1 is an ALT-Specific Therapeutic Vulnerability in Pre-Clinical Glioma Models

**Figure 6.**
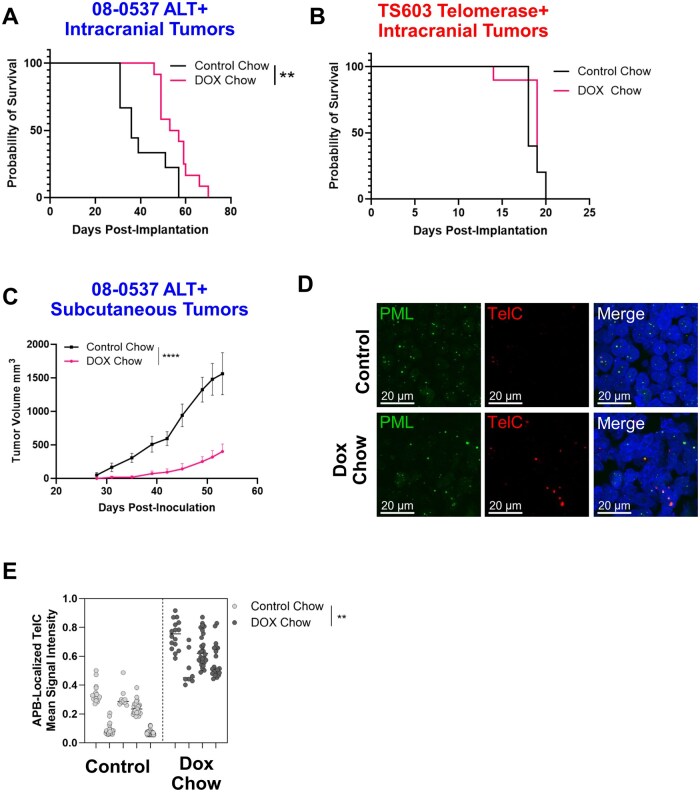
Targeted inhibition of SMARCAL1 is efficacious in ALT+ xenograft models. (A) Survival of nude mice bearing intracranial 08-0537 xenografts with or without inducible depletion of SMARCAL1 via shRNA. Mice were switched to control or doxycycline-containing chow 1-week after intracranial implantation of cells and monitored for the onset of neurological symptoms. A Gehan-Breslow-Wilcoxon test was used to assess differences between control (*n* = 9) and doxycycline chow (*n* = 12) groups. (B) Survival of nude mice bearing intracranial TS603 xenografts treated and analyzed as in (A), with *n* = 10 mice for the control and doxycycline conditions. (C) Longitudinal growth of subcutaneous 08-0537 xenografts expressing doxycycline-inducible shRNA targeting SMARCAL1. Differences in tumor volume between groups was assessed by a mixed-effects analysis with the Šídák method for multiple comparisons, with *n* = 8 mice for control chow and *n* = 7 mice for doxycycline-containing chow groups. (D) Subcutaneous xenograft tissue specimens from nude mice on control or doxycycline-containing chow to induce SMARCAL1-targeting shRNA were analyzed for the intensity of telomeric foci at APBs using an anti-PML antibody and a TelC PNA probe with DAPI counterstaining of nuclei. Images were acquired on a Zeiss 780 upright confocal microscope. (E) Quantitation of mean TelC signal intensity at APBs measured using Cell Profiler software. Statistical differences between experimental conditions were assessed by a nested *t*-test to compare means of control and doxycycline groups.

To investigate whether SMARCAL1 promotes growth of ALT-positive xenografts in vivo, we used our inducible shRNA model in ALT-positive *IDH*-mutant astrocytoma xenografts grown intracranially in nude mice (Figure S9A). After intracranial implantation, mice were switched to control or doxycycline-containing chow and observed for the onset of neurological symptoms. We observed a significant increase in median symptom-free survival in mice with 08-0537 xenografts in which SMARCAL1 had been depleted by RNAi (55 days) relative to the control condition (36 days) (Figure 6A). In contrast, there was no increase in the survival of nude mice bearing orthotopic xenografts of the telomerase-positive cell lines TS603 under the same doxycycline-containing chow conditions (Figure 6B). Doxycycline-induced depletion of SMARCAL1 also significantly inhibited the growth of 08-0537 tumors when grown subcutaneously (Figure 6C), and suppression of 08-0537 cell proliferation was observed in response to SMARCAL1 depletion using 2 distinct SMARCAL1-targeting shRNAs (Figure S9B). Analysis of xenograft tissue from subcutaneous 08-0537 tumors showed that SMARCAL1 suppression caused a significant increase in the intensity of telomeric foci at APBs (Figure 6D-E), consistent with the hyperactivation of the ALT phenotype observed in cell culture studies (Figure 4A-E). Collectively, these results demonstrate that SMARCAL1 is a targetable synthetic lethal vulnerability in ALT-positive gliomas.

## Discussion

In this study, we used a combination of established cancer cell lines and patient-derived glioma models to identify that inhibition of SMARCAL1 is a highly specific therapeutic vulnerability in ATRX-deficient cancer cells that utilize ALT as a mechanism of telomere maintenance. Critically, we found that DNA damage induced by SMARCAL1 depletion was specific to ALT-positive cells, as targeting SMARCAL1 in telomerase-positive glioma cell lines did not induce DNA DSBs (Figures 3F and G, S5D). Moreover, the vast majority of non-ALT cancer cells in the Cancer DepMap dataset showed little to no effect of *SMARCAL1* deletion, while inducible SMARCAL1 depletion by RNAi slowed the proliferation of ALT-positive glioma cells and inhibited the growth of a patient-derived *IDH*-mutant astrocytoma xenografts in nude mice (Figures 2D, S5E, S6A,C). These results suggest that targeted inhibition of SMARCAL1 is efficacious in ALT-positive ATRX-glioma models and nominate SMARCAL1 as a promising synthetic lethal target for anti-cancer drug development.

Published studies from our laboratory and others has shown that rare cases of GBM and osteosarcoma harbor somatic loss-of-function mutations in *SMARCAL1* that contribute to the activation of ALT.^14^^,^^46^^,^^47^ Notably, somatic *SMARCAL1* mutations in adult *TERT* promoter wildtype GBM are mutually exclusive with *ATRX* loss-of-function mutations, supporting the notion that SMARCAL1 and ATRX exhibit a synthetic lethal interaction in gliomas.^14^ In this study, we have shown that SMARCAL1 is a critical mechanism by which cells restrain ALT activity in ATRX-deficient glioma and that depleting SMARCAL1 in this context leads to a hyperactivation of the ALT phenotype, DNA DSBs, and cytotoxicity in a manner that significantly suppresses growth of ALT+ xenografts in mice.

Our analyses of the DepMap CRISPR dataset, including the empirical validation of novel ALT-positive glioma cell lines (Figure S2A-D), revealed that the most specific ALT-associated therapeutic vulnerabilities are the inhibition of SMARCAL1 and FANCM. Both SMARCAL1 and FANCM catalyze the reversal of stalled replication forks and the removal of R-loops,^37^^,^^38^^,^^44^^,^^48^ suggesting that these shared functions may be an Achilles’ heel of ALT-positive cancers. Mechanistically, replication fork reversal and stabilization may compete with ALT by preventing DNA strand breaks at stalled replication forks and thereby suppressing consequent break-induced replication of telomeres.^49^^,^^50^ In our studies, inducible depletion of SMARCAL1 resulted in higher levels of DNA DSBs, increased abundance and intensity of ultrabright telomeric foci, and increased telomeric ssDNA and c-circles. These results are consistent with the induction of a “hyper-ALT” phenotype upon SMARCAL1 depletion and are supported by previous studies that demonstrated FANCM and SMARCAL1 suppress ALT activity, cooperatively regulate the generation of single-stranded telomeric DNA, and that depletion of FANCM in ALT-positive cells leads cell death in a BLM-dependent manner.^8^^,^^9^^,^^12^^,^^18^^,^^51^ In the context of ATRX-deficient cancer cells, our results suggest that moderation of break-induced replication is needed to strike an adaptive balance between replication fork reversal, mediated by SMARCAL1, and break-induced replication of telomeres (Figure S10).

Using patient-derived glioma models, we have shown that inducible inhibition of SMARCAL1 exerts a therapeutic effect by increasing DNA damage, inducing cytotoxicity, and suppressing the growth of ALT-positive xenografts in immune-compromised mice. These results suggest that SMARCAL1 is a bona fide therapeutic target in ATRX-deficient ALT-positive cancers and is the first study to demonstrate efficacy of SMARCAL1 inhibition in glioma models. Importantly, our results show that SMARCAL1 inhibition is likely to be effective independent of the presence of *IDH1/2* mutations, as the effects of SMARCAL1 depletion were observed in both ALT-positive *IDH*-mutant astrocytoma cells and *IDH*-wildtype GBM cells (Figure 2D). However, it is important to note that numerous published studies indicate an important role of *IDH1* mutations and D-2-hydroxyglutarate in modulating homology directed repair (HDR) and inducing heterochromatin-associated replication stress.^52–55^ Therefore, while our results suggest that the presence of an *IDH1* mutation is not required to sensitize cells to SMARCAL1 depletion, we cannot exclude the possibility that *IDH1* mutations and D-2-HG contribute to the efficacy of SMARCAL1 inhibition in ALT-positive cells by increasing DNA replication stress or modulating HDR. It will be important for future studies to evaluate the effect of D-2-HG on the response to SMARCAL1 inhibition, as well as the effect of prior or concurrent treatment with the FDA-approved IDH1/2 inhibitor vorasidenib.

Recently, Leuzzi et al. reported that SMARCAL1 activity is critical for maintaining genomic stability in breast cancer cell lines, and that genetic depletion of SMARCAL1 leads to DNA damage and the activation of cGAS-STING signaling in a manner that potentiates anti-tumor immunity in pre-clinical models of colon cancer.^56^ While it is interesting to consider whether SMARCAL1 inhibition may be a potential strategy to similarly inflame the glioma microenvironment, it is important to note that double-stranded DNA-sensing pattern recognition receptors (eg cGAS-STING) are markedly suppressed in gliomas.^57^ It will therefore be important for future studies to systematically examine the interplay between SMARCAL1-targeting and the activation of anti-tumor immunity in immune competent glioma models. Interestingly, the therapeutic potential of SMARCAL1 and FANCM may expand beyond the scope of ALT-positive cancers. For example, 2 recent studies reported that SMARCAL1 and FANCM display a synthetic lethal interaction due to a redundant function of these enzymes in preventing DNA breakage at simple repeat sequences.^58^^,^^59^ Pan-cancer analyses using data from The Cancer Genome Atlas (TCGA) and Chinese Glioma Genome Atlas (CGGA) have also shown that high SMARCAL1 expression is associated with shorter survival in specific cancer subtypes, including gliomas, lung adenocarcinomas, and liver cancer.^56^^,^^60^ Collectively, these results suggest that SMARCAL1 may be a promising therapeutic target in several cancer types, yet the specific cancer subtypes and genetic contexts that are most vulnerable to SMARCAL1 inhibition remain to be fully elucidated.

In summary, our evidence demonstrates that ATRX-deficient ALT-positive glioma cells display an elevated level of baseline DNA replication stress that renders these tumors highly sensitive to SMARCAL1 depletion in pre-clinical models. SMARCAL1 depletion induces a hyper-ALT phenotype, marked by excessive levels of telomeric ssDNA and ALT activation, which leads to increased DNA damage in G2 phase and mitotic cell death. This study demonstrates that SMARCAL1 is a promising therapeutic target for ALT-positive gliomas and supports small molecule drug development of SMARCAL1 inhibitors. As an ATP-dependent annealing helicase, SMARCAL1 is an inherently druggable enzyme and our results demonstrate that small molecule SMARCAL1 inhibitors have significant potential for the treatment of ATRX-deficient gliomas and other ALT-positive cancers.

## Supplementary Material

noaf300_Supplementary_Data

## Data Availability

Original experimental data and analyses of public datasets are included in the article and Supplementary Materials. Additional information that support the findings of this study or will help others reproduce and test the conclusions of this study are available upon request to the corresponding author.
