# Supplementary material for: SMARCAL1 is a targetable synthetic lethal therapeutic vulnerability in ATRX-deficient gliomas that use alternative lengthening of telomeres
Source: Neuro Oncol. 2026 Jan 10;28(4):895–910. doi: 10.1093/neuonc/noaf300 (PMC13003928; doi:10.1093/neuonc/noaf300)
Supplement: noaf300_Supplementary_Data [file noaf300_supplementary_data.zip › Supplemental Table 1 and 2.docx]

**Supplemental Table 1**

| **ALT-positive and Telomerase-positive Glioma Cell Lines** | | | | | | |
| --- | --- | --- | --- | --- | --- | --- |
| **Cell Line** | **IDH status** | **TERT status** | **ATRX status** | **Diagnosis** | **Grade** | **Telomere Maintenance** |
| TB096 | WT / R132H | WT | Deficient | Astrocytoma-IDH^mut^ | 3 | ALT |
| 08-0537 | WT / R132H | WT | Deficient | Astrocytoma-IDH^mut^ | 4 | ALT |
| 08-0714 | - / R132H | WT | Deficient | Astrocytoma-IDH^mut^ | 4 | ALT |
| D645MG | WT / WT | N.D. | Deficient | PXA | 3 | ALT |
| BT142 | - / R132H | WT | Deficient | Astrocytoma-IDH^mut^ |  | ALT |
| TS603 | WT / R132H | Mutant | Expressing | Oligodendroglioma | 3 | Telomerase |
| 12-0160 | WT / WT | C228T | Expressing | Glioblastoma-IDH^WT^ | 4 | Telomerase |
| 13-0302 | WT / WT | C250T | Expressing | Glioblastoma-IDH^WT^ | 4 | Telomerase |

**Supplemental Table 1. ALT-positive and telomerase-positive glioma cell lines.** List of patient-derived cell lines used in this study and their associated status for genetic alterations involved in glioma diagnosis and telomere maintenance. N.D. indicates not determined.

**Supplemental Table 2**

| **DepMap ID** | **Cell Line Name** | **Primary Disease** | **Lineage** | **ATRX status** | **TP53 status** | **DAXX status** | **ALT status** | **References** |
| --- | --- | --- | --- | --- | --- | --- | --- | --- |
| ACH-000082 | G292 A141B1 | Osteosarcoma | Bone | Wildtype | V274I | DAXX fusion | Positive | Lovejoy et al 2012 |
| ACH-000133 | HS729 | Rhabdomyosarcoma | Soft Tissue | C1595* (deficient) | H179R | Wildtype | Positive | Li et al. 2023 |
| ACH-000341 | SKNFI | Neuroblastoma | PNS | Wildtype | M246R | DAXX low | Positive | Farooqi et al. 2014 |
| ACH-000364 | U2OS | Osteosarcoma | Bone | Exon deletion (deficient) | Wildtype* | Wildtype | Positive | Lovejoy et al 2012 |
| ACH-000410 | SAOS2 | Osteosarcoma | Bone | Deleted (deficient) | deficient | Wildtype | Positive | Lovejoy et al. 2012 |
| ACH-000592 | TM31 | Diffuse Glioma | CNS/Brain | E2281* (deficient) | C238G | Wildtype | Positive | Zimmermann et al. 2022;  This study. |
| ACH-001481 | CHLA90 | Neuroblastoma | PNS | In-frame fusion (deficient) | E286K | Wildtype | Positive | Farooqi et al. 2014 |
| ACH-001526 | HUO9 | Osteosarcoma | Bone | Deficient | CAB39-TP53 fusion | Widltype | Positive | Yost et al. 2019 |
| ACH-001610 | NP5 | Diffuse Glioma | CNS/Brain | R2028* | R273C, R175H | Wildtype | Positive | This study |
| ACH-001715 | CAL72 | Osteosarcoma | Bone | Exon deletion (deficient) | TP53-P2RX5 fusion | Wildtype | Positive | Yost et al. 2019 |
| ACH-002922 | SKNMM | Neuroblastoma | PNS | K1367* (deficient) | C135F | Wildtype | Positive | Zeineldin et al. 2020 |
|  |  |  |  |  |  |  |  |  |
| ACH-002806 | TB096 | Diffuse Glioma | CNS/Brain | R781* (deficient) | G245V | Wildtype | Positive | This study |
|  | *U2OS harbors a PPM1D truncating mutation | | |  |  |  |  |  |

**Supplemental Table 2. ALT-positive cell lines in the Cancer Dependency Map dataset.** List of *ATRX*/*DAXX* altered ALT-positive cell lines within the DepMap dataset used for the analysis of gene dependencies compared to all other cell lines in the dataset. All cell lines included have been empirically validated for the presence of ALT biomarkers in this study or in published studies.
