# Supplementary material for: SMARCAL1 is a targetable synthetic lethal therapeutic vulnerability in ATRX-deficient gliomas that use alternative lengthening of telomeres
Source: Neuro Oncol. 2026 Jan 10;28(4):895–910. doi: 10.1093/neuonc/noaf300 (PMC13003928; doi:10.1093/neuonc/noaf300)
Supplement: noaf300_Supplementary_Data [file noaf300_supplementary_data.zip › Supplemental Figure Legends.docx]

**Supplemental Figure Legends**

**Supplemental Figure 1. Characterization of ALT-positive glioma cell lines.** (A) Confocal microscopy images showing the presence of APBs in ALT-positive glioma cell lines and the co-localization of γH2AX foci within APBs. Cells were grown on chamber slides and stained by IF-FISH using a TelC-AlexaFluor-647 probe and anti-PML and anti- γH2AX primary antibodies. Primary antibodies were detected via anti-mouse-IgG-AlexaFluor594 and anti-rabbit-IgG-AlexaFluor488 secondary antibodies and nuclei were counterstained with DAPI. Images were acquired on a Zeiss 780 upright confocal microscope under 63x oil immersion.(B) BT142 IDH-mutant astrocytoma cells were stained with a TelG PNA probe under native conditions to detect single-stranded C-rich telomeric DNA according to the ssTeloC assay. Positive staining is one indicator of the presence of ALT in these cells.

**Supplemental Figure 2. Identification of ALT-positive glioma cell lines in the DepMap dataset.** (A) IF-FISH staining of *IDH*-wildtype GBM cells TM-31 indicates the presence of APBs, indicated by co-localized TelC and PML, as well as the presence of ssTeloC foci. (B) IF-FISH staining of *IDH*-wildtype GBM cells NP5 cells for PML and TelC demonstrates the presence of APBs in these cells. (C) Western blot analysis of NP5 and TM-31 whole cell lysates demonstrates that these cell lines are deficient for full-length ATRX protein. LN229 GBM cells are included as a positive control for ATRX expression. (D) TM-31 cells were processed for the ALT telomere synthesis assay (ATSA). Cells were enriched in G2 phase by treatment with Ro-3306 (5μM) for 24 hours, pulse labeled with EdU (10 μM) for 2 hours, and then processed for IF-FISH with TelC and PML. The co-localization of EdU foci within APBs demonstrates de novo telomere synthesis in this cell line.

**Supplemental Figure 3. Gene dependencies in ALT-positive cancer cell lines in the DepMap CRISPR screening dataset.** (A) TERF2IP, (B) CENPX, (C) FANCF, (D) ATR, and (E) ETAA1 showed greater detrimental effects upon gene deletion via sgRNA in ALT-positive cell lines versus all other cell lines in the DepMap dataset.

**Supplemental Figure 4. SMARCAL1 localizes to APBs in ALT-positive glioma cell lines.** (A) Western blot detection of ATRX and SMARCAL1 protein expression in a panel of telomerase+ and ALT-positive glioma cell lines. All ALT-positive lines are ATRX-deficient and exhibit APBs. (B) Confocal microscopy images of BT-142 *IDH*-mutant astrocytoma cells stained by IF-FISH using a TelC PNA probe and SMARCAL1 and PML antibodies. SMARCAL1 foci localize to APBs in APB-positive nuclei. Images were acquired on a Zeiss 780 upright confocal microscope under 63x oil immersion. (C) NP5 *IDH*-wildtype GBM cells analyzed as in (B). (D) D645MG pleiomorphic xanthoastrocytoma cells analyzed as in (B).

**Supplemental Figure 5. SMARCAL1 depletion leads to DNA DSBs and suppresses proliferation in ALT-positive glioma cell lines.** (A) Western blot analysis γH2AX induction following doxycycline-inducible (1 μg/ml) depletion of SMARCAL1 via shRNA over a 144-hour timecourse in ALT-positive TB096 cells. Differences between groups were assessed using an unpaired two-tailed t-test. (B) Experiment performed and analyzed as in (A) in TM-31 GBM cells. (C) Western blot analysis γH2AX induction following doxycycline-inducible depletion of SMARCAL1 via shRNA over a 144-hour timecourse in ALT-positive D645MG cells. Differences between conditions were analyzed using a one-way ANOVA and Dunnett’s multiple comparisons test. (D) Western blot analysis of relative γH2AX levels in LN229 cells following inducible depletion of SMARCAL1 for 96 hours. Differences between groups were assessed using an unpaired two-tailed t-test**.** (E) TB096 cells with doxycycline-inducible non-targeting or SMARCAL1-targeting shRNA were seeded in 6 well plates (10^4^ cells per well). After 3 days of culture, 1 μg/ml doxycycline was added to culture media and cells were allowed to proliferate for two weeks. Confluence was tracked over time with the IncuCyte live cell imaging machine. Data represent n=3 technical replicates and were analyzed using an unpaired two-tailed t-test. (F) TB096 cells transduced with doxycycline-inducible non-targeting or SMARCAL1-targeting shRNA were treated with or without doxycycline for 6-days, after which the extent of nuclear DNA DSBs was assessed by γH2AX confocal IF microscopy. Data represent two independent experiments quantitated from 3x3 tiled fields and analyzed via an unpaired Mann-Whitney test relative to the minus-doxycycline condition within each shRNA-expressing line.

**Supplemental Figure 6. SMARCAL1 suppresses ALT-associated phenotypes in ALT-positive glioma cel lines** (A) TB096 transduced with doxycycline-inducible SMARCAL1-targeting shRNA were treated with doxycycline (1 μg/ml) for 96 or 144 hours, after which genomic DNA was isolated and analyzed via c-circle assay. (B) D645MG cells with doxycycline-inducible SMARCAL1-targeting shRNA treated and analyzed for the abundance of c-circles as in (A). (C) The ssTeloC assay was performed on TM-31 cells after depletion of SMARCAL1 via shRNA for 96 or 144 hours. Nuclear single-stranded c-rich DNA was detected using a TelG PNA probe conjugated to Alexa-Fluor-488, nuclei were counterstained with DAPI, and images were acquired under oil immersion using a 63x objective on a Zeiss 780 upright confocal microscope. (D) Images acquired as described in (C) were analyzed using Cell Profiler software to measure integrated nuclear ssTeloC intensity. Differences between groups was assessed via a one-way ANOVA and Tukey’s multiple comparisons test.

**Supplemental Figure 7. Depletion of SMARCAL1 leads to extensive DNA damage in G2 and perturbs cell cycle progression.** (A) TM-31 GBM cells stably expressing an inducible SMARCAL1-targeting shRNA vector were treated with doxycycline (1 μg/ml) for 6 days, pulse labeled with EdU (10 μM) for 30 minutes, and then processed for immunofluorescence. γH2AX staining intensity was quantitated on a nucleus-by-nucleus basis in G1 (EdU^-^ / Cyclin A2^-^), S-phase (Edu^+^), and G2 (Edu^-^ / Cyclin A2^+^) using Cell Profiler software. (B) Cell cycle analysis of TB096 cells treated with doxycycline for 6 days to inducibly deplete SMARCAL1. Differences between groups was assessed by an unpaired two-tailed t-test and correction for multiple comparisons using the Holm-Šídák method. (C) Cell cycle analysis of TB096 cells treated with doxycycline for 6 days to inducibly deplete SMARCAL1. Differences between groups were assessed by an unpaired two-tailed t-test and correction for multiple comparisons using the Holm-Šídák method. (D) TM-31 cells treated and analyzed as in (C).

**Supplemental Figure 8. SMARCAL1 depletion causes mitotic cell death in ALT-positive glioma cells.** (A) Representative DAPI-stained nuclear morphologies observed in TB096 IDH-mutant astrocytoma cells after 6-days depletion of SMARCAL1 via doxycycline-inducible shRNA. (B-D) Quantitation of nuclear morphologies from DAPI-stained nuclei imaged under 63x oil immersion. (E) Quantitation of the percentage of nuclei exhibiting 2 or more nuclear abnormalities quantitated as in B-D. A chi-square test was used to assess differences in proportions across groups in B (p=.0002), C (p<.0001), D (not calculated, infrequent events), and E (p<.0001).

**Supplemental Figure 9. Validation of SMARCAL1 depletion for in vivo studies and effects on DNA DSBs.** (A) 08-0537 cells expressing a SMARCAL1-targeting doxycycline-inducible shRNA were treated with 1 μg/ml doxycycline in cell culture media for 6-days. Cells were lysed and processed for western blot analysis of SMARCAL1 protein levels. (B) ALT+ 08-0537 cells expressing one of two distinct SMARCAL1-targeting shRNAs were seeded in 96 well plates and allowed to proliferate in the absence or presence of doxycycline for 10-days. Cell viability was measured using CellTiterGlo and values presented relative to the minus-doxycycline controls (represented by dashed line). Differences between treatment conditions were analyzed using a unpaired two-tailed t-test.

**Supplemental Figure 10**. **SMARCAL1 restricts excessive ALT activity to maintain genomic stability**. (A) ATRX-deficiency in ALT-positive glioma cells leads to higher levels of baseline replication stress relative to telomerase positive cells. Elevated levels of telomeric replication stress leads to increased frequency of stalled replication forks stalling, which can be reversed and re-started by SMARCAL1 (left) or lead to break-induced replication and telomere extension (right). (B) When SMARCAL1 activity is lost, ALT activity is hyper-activated in the absence of SMARCAL1-mediated fork reversal, thus leading to excessive break-induced telomere replication, intolerable replication stress, high levels of telomeric DNA DSBs, and mitotic cell death. Image created using Biorender.com.
