# Supplementary material for: SMARCAL1 is a targetable synthetic lethal therapeutic vulnerability in ATRX-deficient gliomas that use alternative lengthening of telomeres
Source: Neuro Oncol. 2026 Jan 10;28(4):895–910. doi: 10.1093/neuonc/noaf300 (PMC13003928; doi:10.1093/neuonc/noaf300)

Supplemental Figure 1

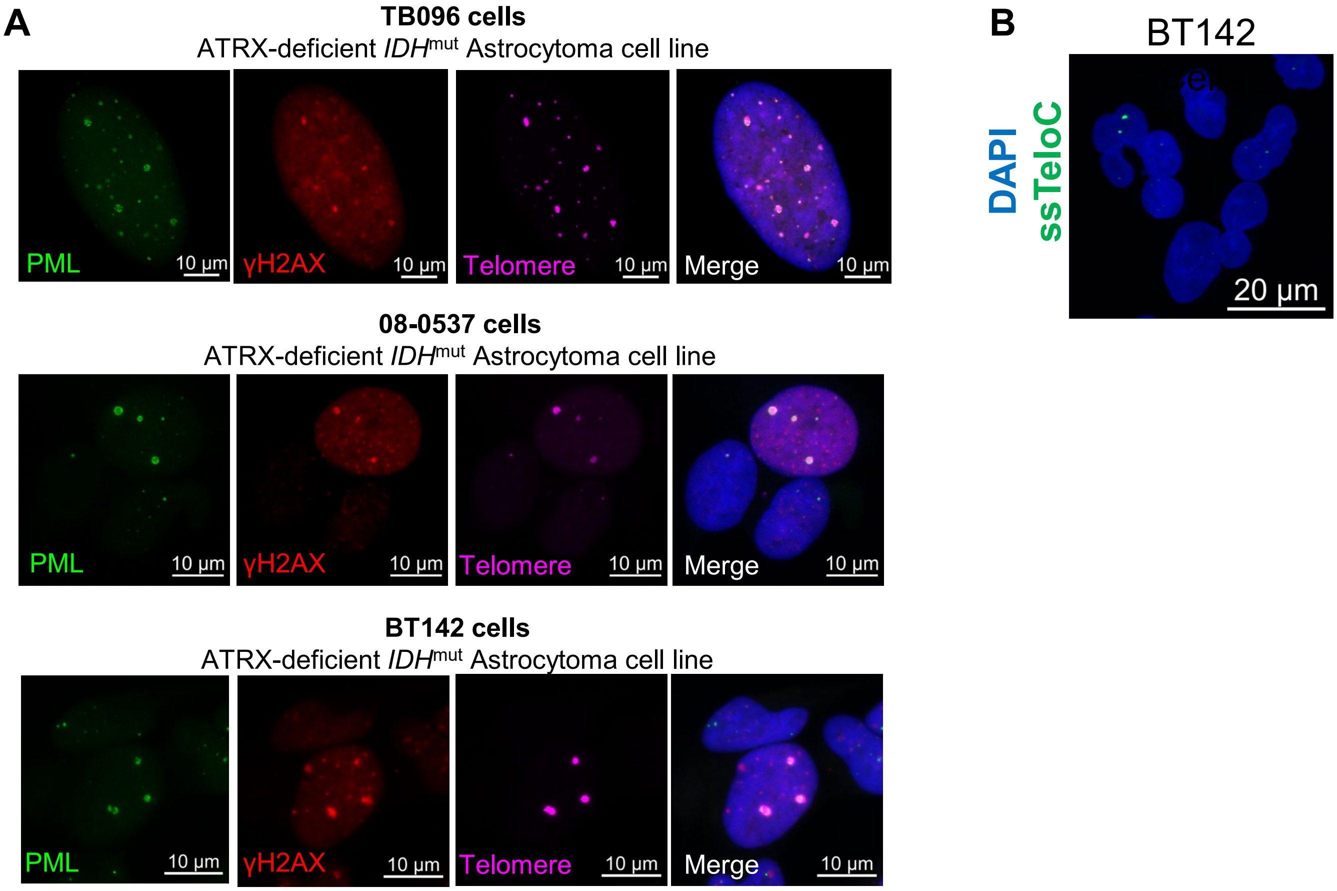

Supplemental Figure 2

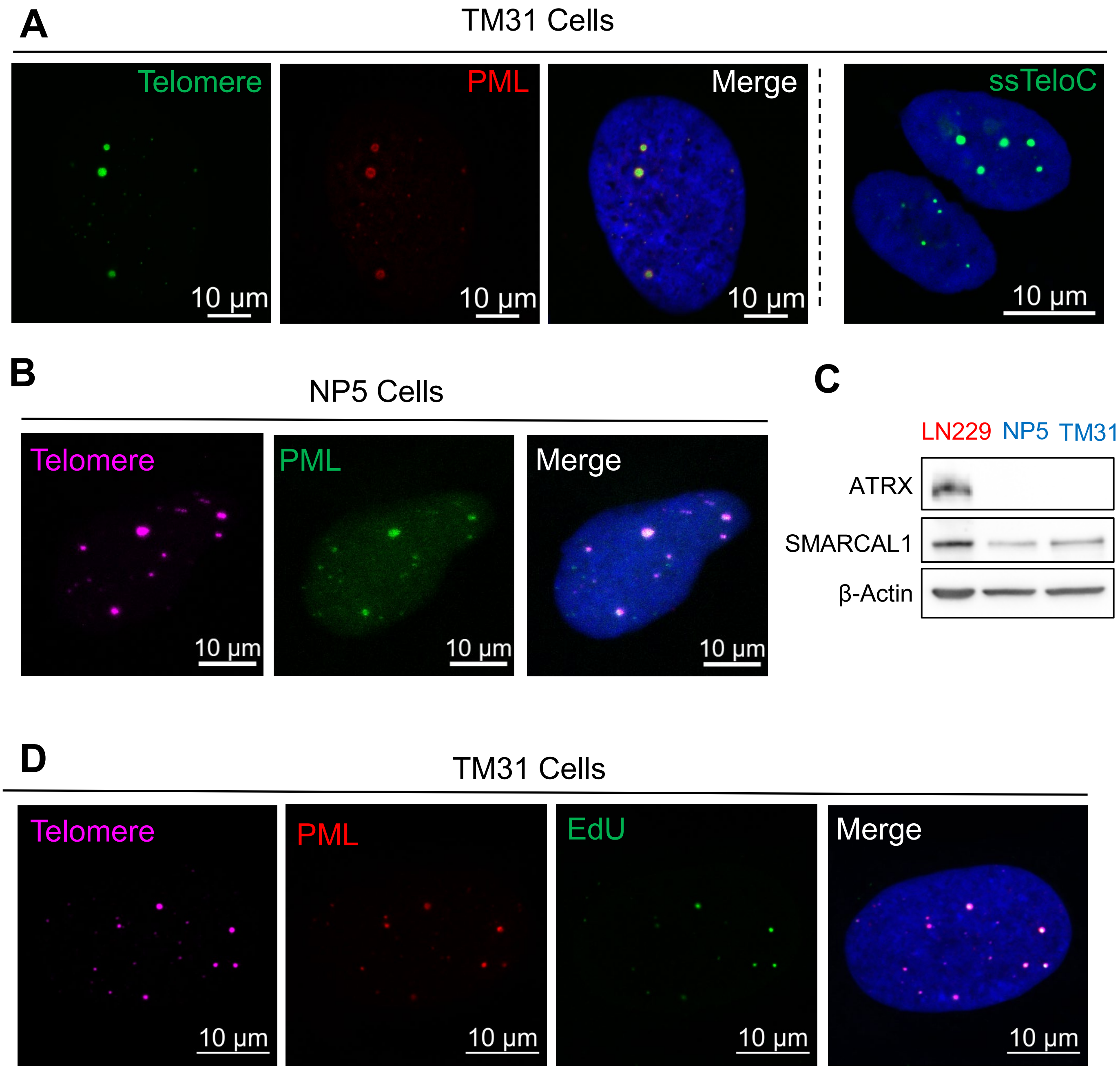

Supplemental Figure 3

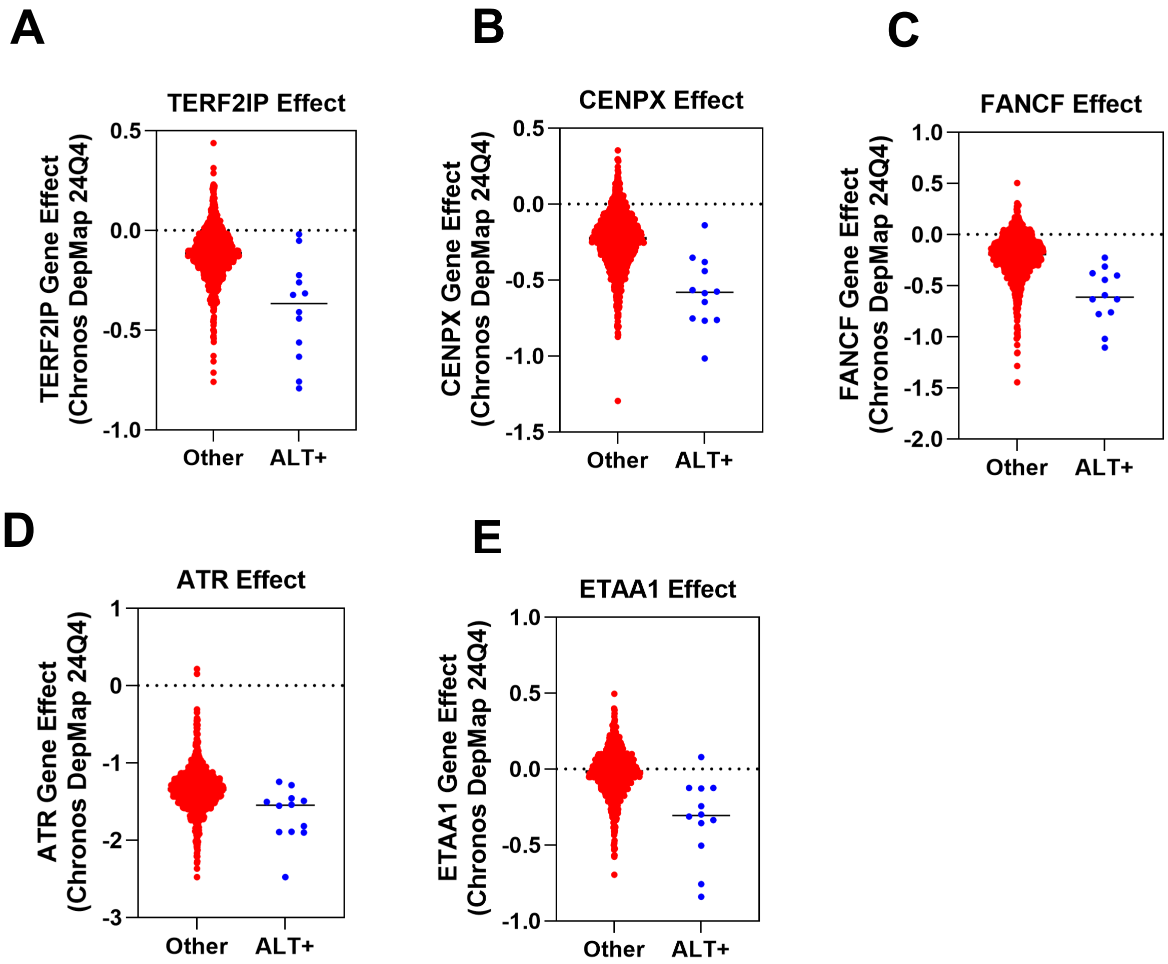

Supplemental Figure 4

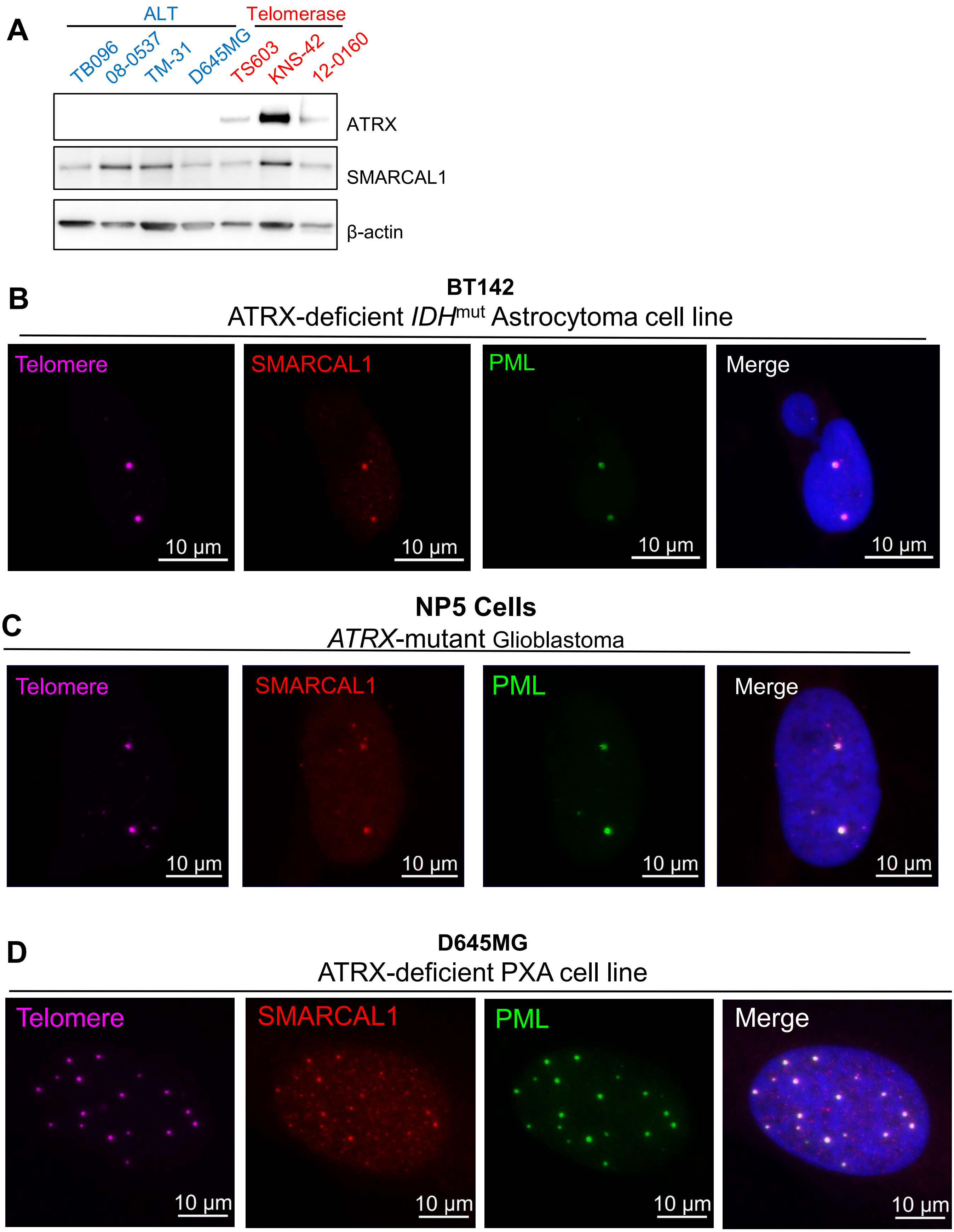

Supplemental Figure 5

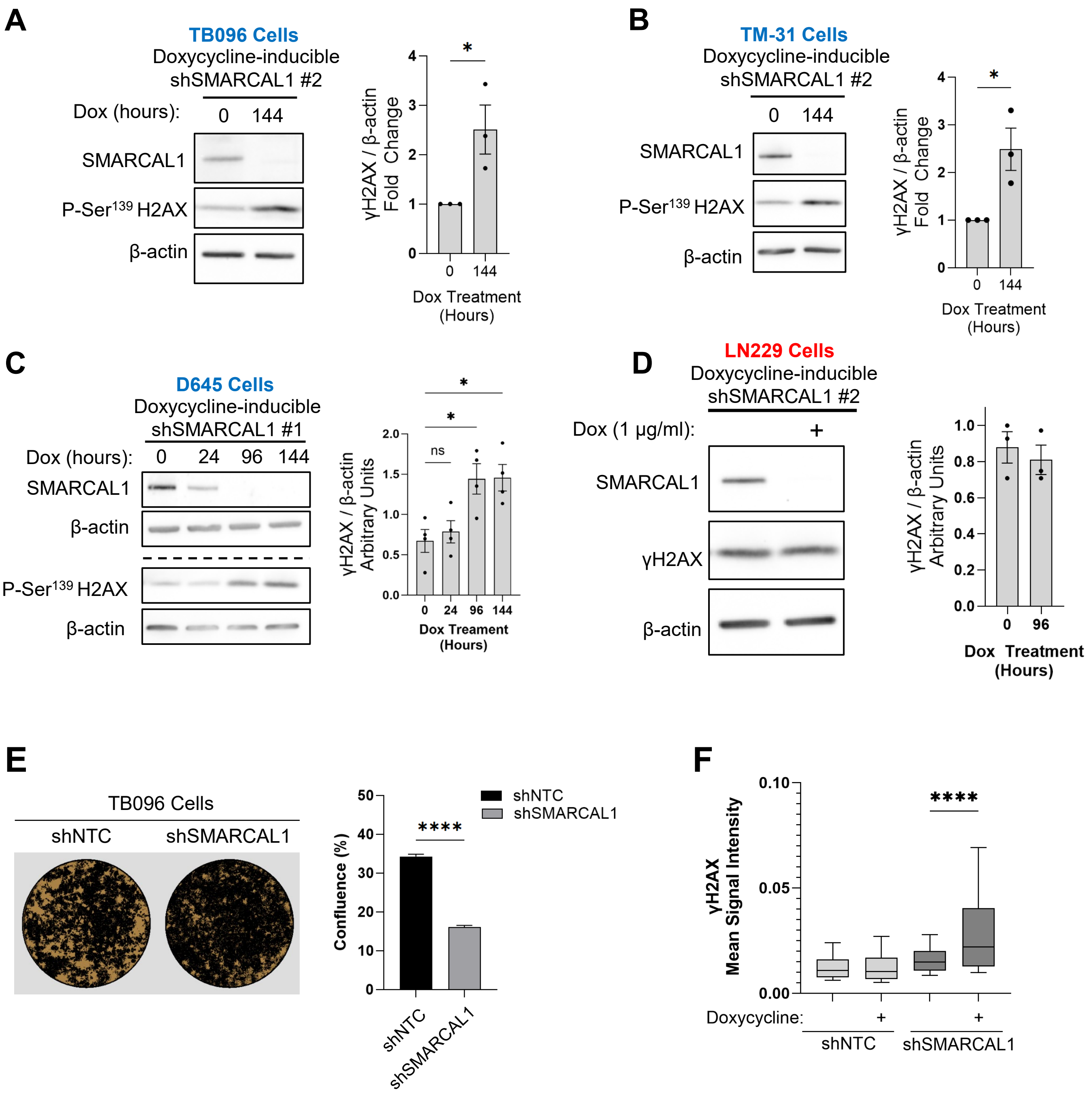

Supplemental Figure 6

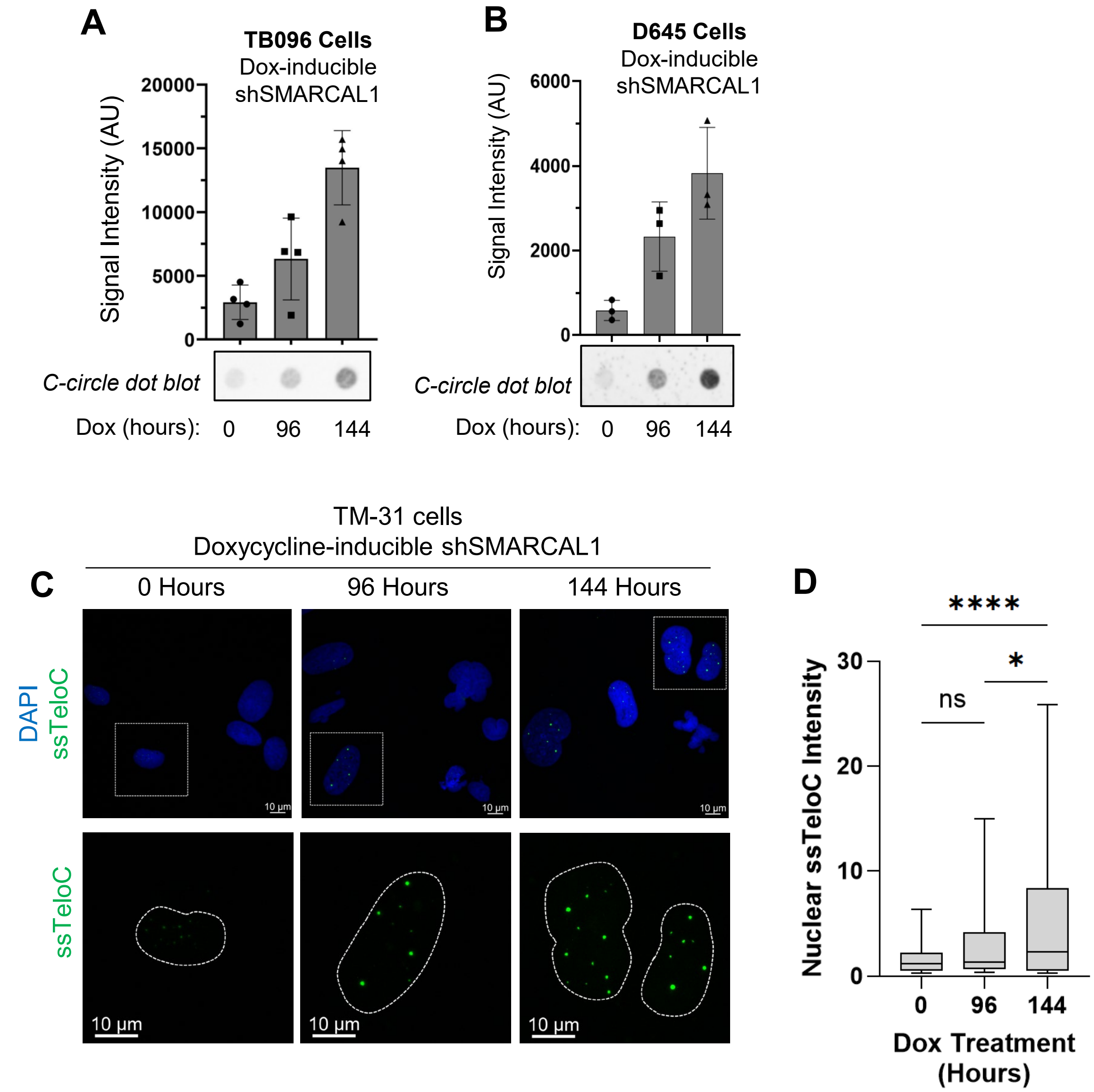

Supplemental Figure 7

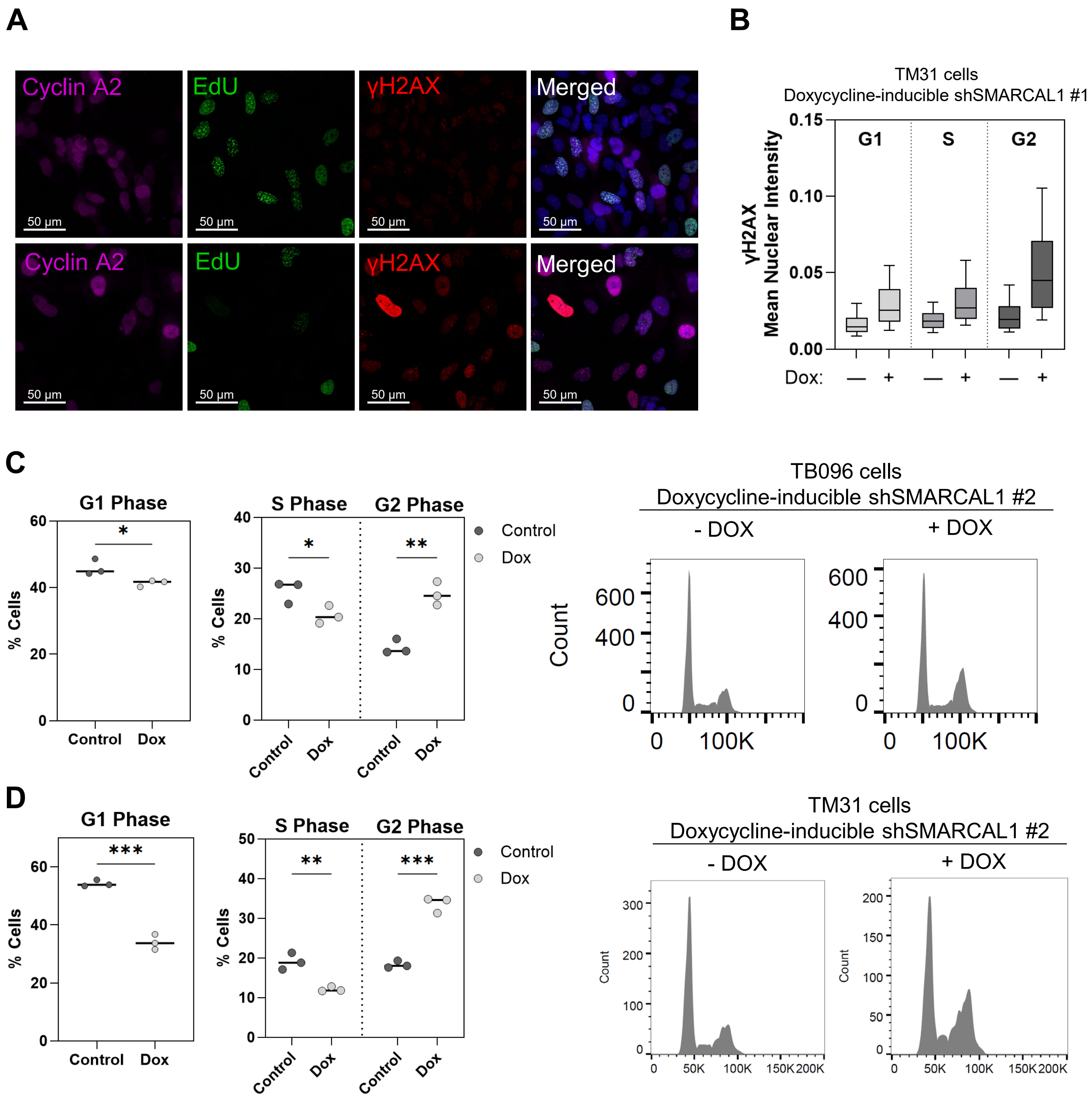

Supplemental Figure 8

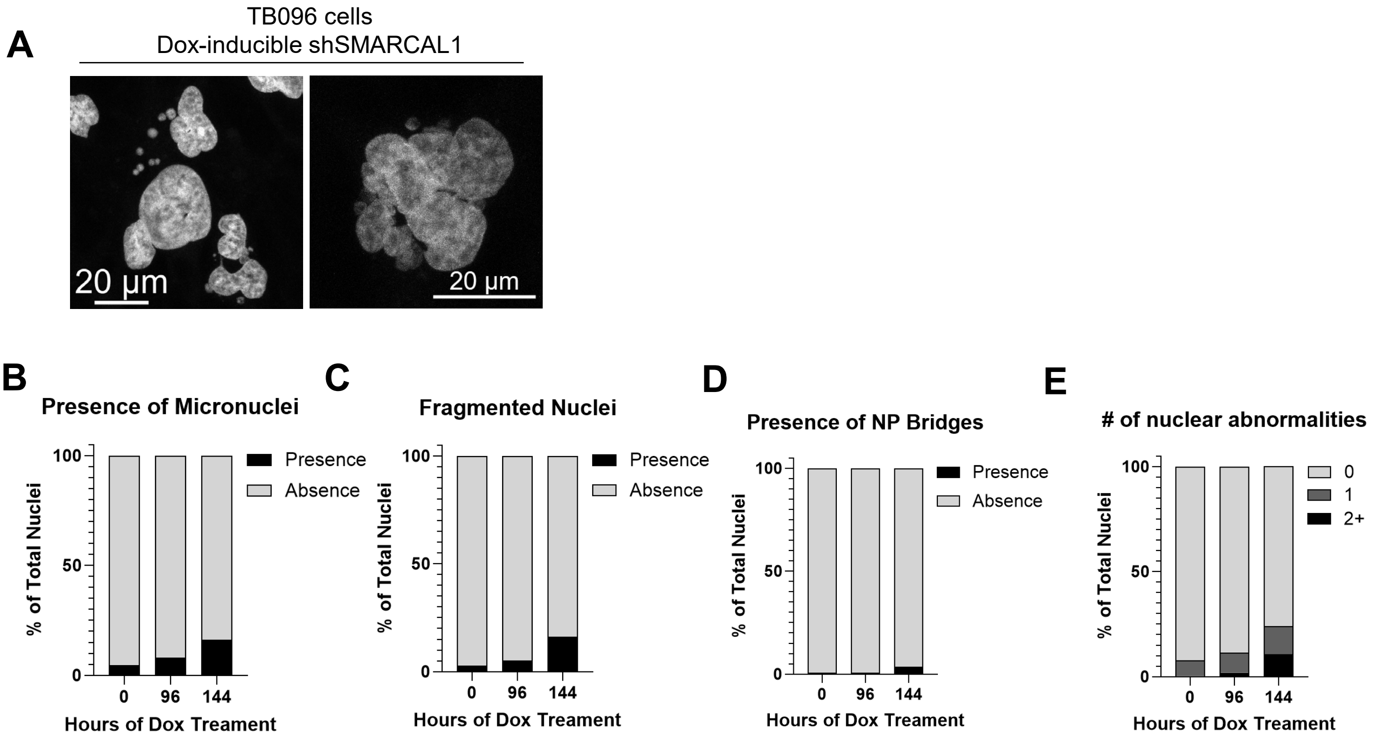

Supplemental Figure 9

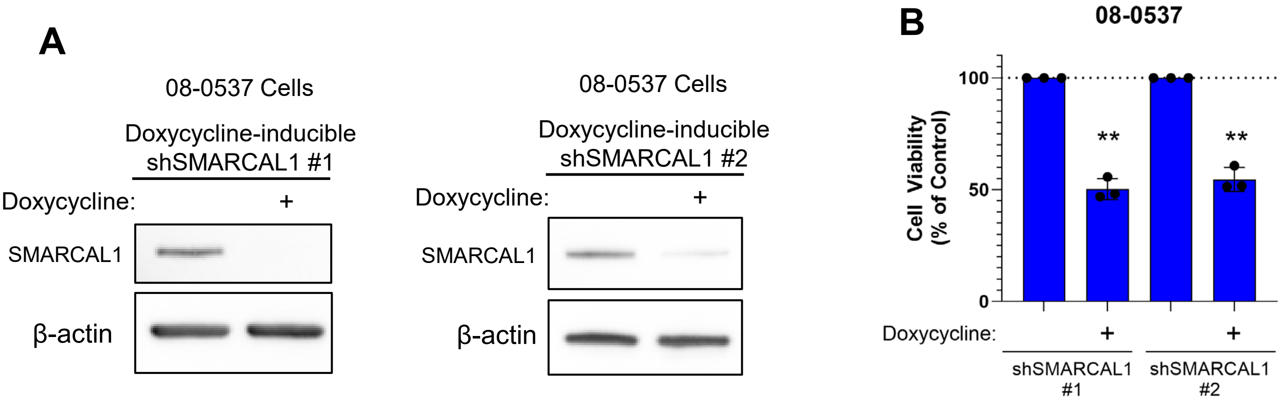

Supplemental Figure 10

A

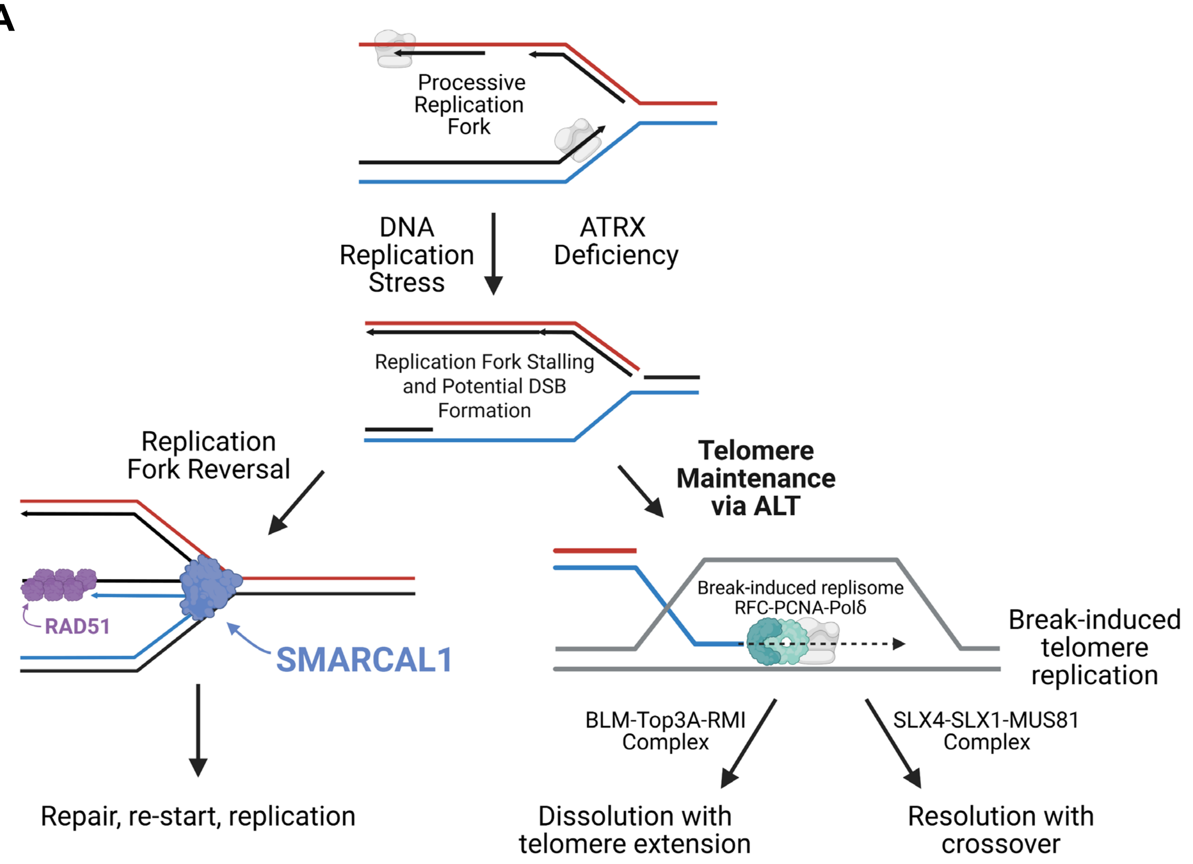

B

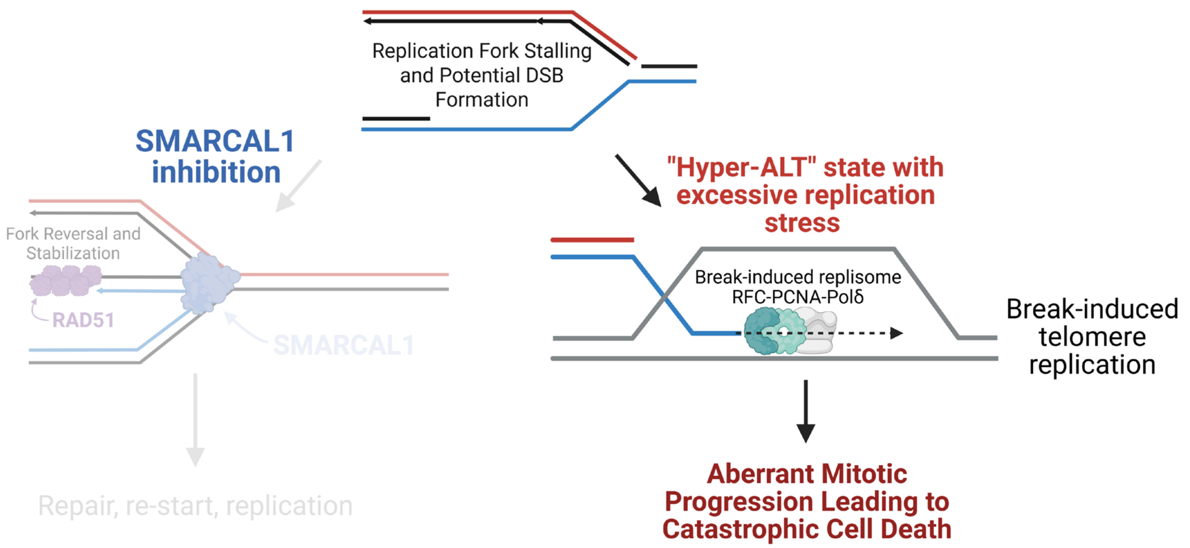

Supplement: noaf300_Supplementary_Data [file noaf300_supplementary_data.zip › Supplemental Figures.pdf]
