# Supplementary material for: SMARCAL1 is a targetable synthetic lethal therapeutic vulnerability in ATRX-deficient gliomas that use alternative lengthening of telomeres
Source: Neuro Oncol. 2026 Jan 10;28(4):895–910. doi: 10.1093/neuonc/noaf300 (PMC13003928; doi:10.1093/neuonc/noaf300)
Supplement: noaf300_Supplementary_Data [file noaf300_supplementary_data.zip › Supplemental Materials and Methods.docx]

**Supplemental Materials and Methods**

**Table 1. Plasmids and Sequences**

| **Reagent** | **Sequence** | **Vendor/Source** |
| --- | --- | --- |
| pLKO Tet-on shRNA non-targeting | CCTAAGGTTAAGTCGCCCTCGCTCGAGCGAGGGCGACTTAACCTTAGG | Duke |
| pLKO Tet-on shSMARCAL1 #1 | GGAACTCATTGCAGTGTTTAACTCGAGTTAAACACTGCAATGAGTTCC | Duke |
| pLKO Tet-on shSMARCAL1 #2 | gctttgaccttcttagcaagtCTCGAGacttgctaagaaggtcaaagc | Duke |

**Table 2. Western Blot Antibodies**

| **Target** | **Vendor/Source** | **Catalog #** | **Dilution** | **Notes** |
| --- | --- | --- | --- | --- |
| SMARCAL1 | Cell Signaling | 44717 | 1:1000 | 5% BSA Blocking Buffer |
| ATRX | Cell Signaling | 10321 | 1:1000 | Protein-Free Blocking Buffer |
| γH2AX | Cell Signaling | 80312 | 1:1000 | Protein-Free Blocking Buffer |
| Beta-actin | Cell Signaling | 3700 | 1:10000 | Protein-Free Blocking Buffer |

**Table 3. IF-FISH Antibodies and Probes**

| **Target** | **Vendor/Source** | **Catalog #** | **Dilution** |
| --- | --- | --- | --- |
| SMARCAL1 | Santa Cruz | SC-166209 | 1:100 |
| DAXX | Cell Signaling | 4533 | 1:100 |
| PML | Cell Signaling | 69789 | 1:500 |
| PML | Santa Cruz | SC-966 | 1:100 |
| γH2AX | Cell Signaling | 80312 | 1:100 |
| pCHK1 Ser^317^ | Cell Signaling | 12302 | 1:500 |
| TelC-AlexaFluor-647 | PNA Bio | F1013 | 1:2000 |
| TelC-AlexaFluor-488 | PNA Bio | F1004 | 1:2000 |
| TelG-AlexFluor-488 | PNA Bio | F1008 | 1:2000 |
| Anti-rabbit-AlexaFluor-488 | Invitrogen | A11008 | 1:100 |
| Anti-mouse-AlexaFluor-594 | Invitrogen | A11005 | 1:100 |
| RNase HII-A | Santa Cruz | SC-515475 | 1:50 |
| Cyclin A2 | Cell Signaling | 67955 | 1:800 |
